# Comprehensive Characterization of Phytochemical Composition, Membrane Permeability, and Antiproliferative Activity of *Juglans nigra* Polyphenols

**DOI:** 10.3390/ijms25136930

**Published:** 2024-06-25

**Authors:** Rita Osztie, Tamás Czeglédi, Sarah Ross, Bence Stipsicz, Eszter Kalydi, Szabolcs Béni, Imre Boldizsár, Eszter Riethmüller, Szilvia E. Bősze, Ágnes Alberti

**Affiliations:** 1 Department of Pharmacognosy, Semmelweis University, Üllői út 26, 1085 Budapest, Hungary; osztie.rita@stud.semmelweis.hu (R.O.); czegledi.tamas@semmelweis.hu (T.C.); boldizsar.imre@semmelweis.hu (I.B.); riethmuller.eszter@semmelweis.hu (E.R.); 2 Department Pharmaceutical Biology, Institute for Drug Discovery, University of Leipzig, Eilenburger Str. 14, 04317 Leipzig, Germany; sarah.ross@studserv.uni-leipzig.de; 3 Institute of Biology, Doctoral School of Biology, ELTE Eötvös Loránd University, Pázmány Péter sétány 1/C, 1117 Budapest, Hungary; stipsicz@student.elte.hu; 4 HUN-REN-ELTE Research Group of Peptide Chemistry, Hungarian Research Network, ELTE Eötvös Loránd University, Pázmány Péter sétány 1/A, 1117 Budapest, Hungary; szilvia.bosze@ttk.elte.hu; 5 Institute of Organic Chemistry, Semmelweis University, Hőgyes Endre u. 7., 1092 Budapest, Hungary; kalydi.eszter@semmelweis.hu; 6 Institute of Chemistry, ELTE Eötvös Loránd University, Pázmány Péter sétány 1/A, 1117 Budapest, Hungary; szabolcs.beni@ttk.elte.hu; 7 Department of Plant Anatomy, Institute of Biology, ELTE Eötvös Loránd University, Pázmány Péter sétány 1/C, 1117 Budapest, Hungary; 8 Department of Genetics, Cell- and Immunobiology, Semmelweis University, Nagyvárad tér 4., 1089 Budapest, Hungary

**Keywords:** *Juglans nigra*, black walnut, UHPLC-MS/MS, PAMPA-BBB, PAMPA-GI, juglone

## Abstract

The aim of our study was the detailed polyphenol profiling of *Juglans nigra* and the characterization of the membrane permeability and antiproliferative properties of its main phenolics. A total of 161 compounds were tentatively identified in *J. nigra* bark, leaf, and pericarp extracts by ultrahigh-performance liquid chromatography–high-resolution tandem mass spectrometry (UHPLC-HR-MS/MS). Eight compounds including myricetin-3-*O*-rhamnoside (**86**), quercetin-3-*O*-rhamnoside (**106**), quercetin-3-*O*-xyloside (**74**), juglone (**141**), 1,2,3,4-tetrahydro-7,8-dihydroxy-4-oxonaphthalen-1-yl-6-*O*-galloyl-glucoside (**92**), ellagic acid (**143**), gallic acid (**14**), and ethyl gallate (**58**) were isolated from *J. nigra* pericarp. The in vitro antiproliferative activity of the isolated compounds was investigated against three human cancer cell lines, confirming that juglone (**141**) inhibits cell proliferation in all of them, and has similar activity as the clinical standards. The permeability of the isolated compounds across biological membranes was evaluated by the parallel artificial membrane permeability assay (PAMPA). Both juglone (**141**) and ethyl-gallate (**58**) showed positive results in the blood–brain-barrier-specific PAMPA-BBB study. Juglone (**141**) also possesses log*Pe* values which indicates that it may be able to cross both the GI and BBB membranes via passive diffusion.

## 1. Introduction

Black walnut (*Juglans nigra* L., Juglandaceae) is a tall deciduous tree with a typically dark grey or brownish bark that is deeply split into narrow ridges. It can be distinguished from the extensively cultivated English walnut (*Juglans regia* L.) based on the size and morphology of their leaves. The larger leaves of black walnut are composed of a higher number of lanceolate or ovate–lanceolate and serrate leaflets as compared to the common walnut [1]. The tree is native to the central and eastern regions of North America and was introduced to Europe in the 16th or 17th centuries, where it now grows wild especially in Eastern and Central Europe. Black walnut is cultivated as an ornamental tree and for timber production, and its seeds are also consumed [2,3].

*Juglans* species are rich sources of bioactive natural products: naphthoquinones, tetralones, and naphthalenyl derivatives, diarylheptanoids, hydrolysable tannins, flavonoids, phenolic acids, triterpenes, and sterols have also been reported for the genus [4,5]. Among the constituents attracting the greatest interest are the ones exerting antitumor activities. Juglone (5-hydroxy-1,4-naphthalenedione), the allelopathic naphthoquinone constituent of *Juglans* species, has been shown to induce DNA damage and apoptosis [6,7,8]. Reactive oxygen species (ROS) contribute substantially to the antiproliferative activity of juglone [9,10,11]. Cytotoxic activity of other naphthoquinones isolated from walnut species [12,13] or that of their synthetic derivatives has also been reported [14]. Diarylheptanoids, mostly biaryl-type and diarylether-type macrocyclic representatives, have also been described in *Juglans* species [15,16]. Among them, galeon isolated from *J. mandshurica* [17] as well as juglanin A and B from *J. regia* [18], and more recently jugsigin A from *J. sigillata* [19], have been shown to exhibit cytotoxic effects against human cancer cells.

Lin and their coworkers investigated the metabolite profile of black walnut cultivars; they characterized flavonoids, phenolic acids, and tannins in kernel extracts [20,21,22]. Antora et al. quantified vitamins, minerals, and amino acids in black walnut [23]. Occurrence of caffeoylquinic acids and flavonoid glycosides in the seeds [24] as well as in leaf [25,26] and bark [27,28] samples has also been reported. Additionally, the α-tetralone (−)-regiolone, and eight volatile 1,4-naphthoquinones such as juglone, hydrojuglone, plumbagin, and methylplumbagin have been isolated from the fruits [29,30,31]. 

Kernel extracts from different black walnut cultivars showed antibacterial activity [32]. Green husk extracts showed significant antifungal activity in female rats [33], while extracts prepared by supercritical fluid extraction have exerted antioxidant activity [34]. The bark extract showed neuroprotective activity in a rat model of cerebral ischemia by normalizing mitochondrial function [35]. *J. nigra* leaf extract has also shown antinociceptive activity in rats [36]. Ho et al. also studied the anticancer activities of compounds from *J. nigra* kernels. Pentagalloyl-glucose and quercetin-3-*O*-glucoside inhibited the growth of A549 human lung adenocarcinoma cells. However, the authors did not isolate the constituents from the extract for unambiguous identification [37].

Nevertheless, the comprehensive screening of the phenolic profile of *J. nigra* has not been performed. Therefore, the aim of this study was the phytochemical screening of *J. nigra* leaf, bark, and pericarp samples. To extract a wide range of polyphenolic compounds, we aimed to prepare extracts with solvents of different polarity. For the identification of polyphenols ultrahigh-performance liquid chromatography coupled to diode-array detection and electrospray ionization tandem mass spectrometry (UHPLC-DAD-ESI-MS/MS) was used. Our further objective was to reveal the constituents of black walnut pericarp that might contribute to its biological effects. Therefore, we aimed to isolate representative components and investigate their in vitro antiproliferative activities in various human cancer cell lines. Additionally, we aimed to evaluate the compounds’ ability to permeate biological membranes using parallel artificial membrane permeability assays for the gastrointestinal tract and the blood–brain barrier (PAMPA-GI and PAMPA-BBB).

## 2. Results

### 2.1. Qualitative Analysis of Juglans nigra Polyphenols by UHPLC-DAD-ESI-MS/MS

**Figure 1:**
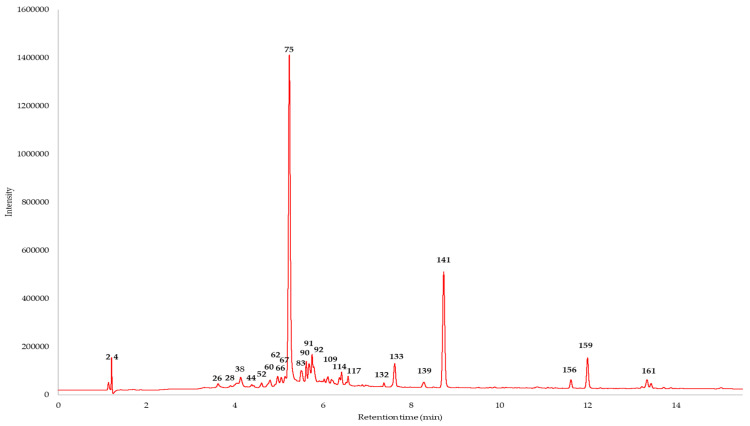
UHPLC-DAD chromatogram of the ethyl acetate extract of *J. nigra* pericarp (max plot). Compound numbers refer to Table 1.

UHPLC-DAD-ESI-MS/MS was used to evaluate the phenolic profile of the black walnut leaf, bark, and pericarp extracts. We performed the tentative characterization of 161 compounds: we compared the retention times, UV spectra, and mass spectra of the detected constituents with the literature data; the results are presented in Table 1. A representative UHPLC-DAD chromatogram of *J. nigra* pericarp ethyl acetate extract is shown in Figure 1, while chromatograms of all extracts (chloroform, ethyl acetate, and methanol extracts of leaf, bark, and pericarp samples) are shown in Supplementary Figures S1–S3.

#### 2.1.1. Characterization and Analysis of Flavonoids

In the analyzed samples, the dominating flavonol derivatives occurred mainly in their glycosidic form. During the collision-induced dissociation (CID) of flavonoid glycosides, cleavage of a hexose, a deoxyhexose, a pentose, or a hexuronose sugar moiety resulted in neutral losses of 162, 146, 132, or 176 Da, respectively. The following molecular and fragment ions were observed for the aglycones: [Y_0_]^−^ at *m*/*z* 285, 301, and 317, [Y_0_−H]^−^ at *m*/*z* 284, 300, and 316 for kaempferol, quercetin, and myricetin, respectively. The glycosylation site of flavonol-3-*O*-glycosides could also be presumed. They favor the homolytic cleavage of the saccharide moiety in negative ionization mode. Therefore, the relative abundance of the radical aglycone ion [Y_0_−H]^−•^ was higher in their mass spectra than that of the aglycone anion [Y_0_]^−^ [73,74,75]. Peaks **90** and **77** displayed their [M−H]^−^ ions at *m*/*z* 463 and 479, respectively, and the [M−H−162]^−^ ions at *m*/*z* 301 (quercetin−H)^−^ and *m*/*z* 317 (myricetin−H)^−^. Therefore, **90** and **77** were identified as quercetin-3-*O*-hexoside and myricetin-3-*O*-hexoside, respectively. Flavonol-3-*O*-deoxyhexoside derivatives (**120**, **106**, **86**) were characterized similarly [36].

Compounds **116** and **117** were identified as myricetin-3-*O*-galloyl-deoxyhexoside isomers as they exhibited the [M−H]^−^ ion at *m*/*z* 615 and fragments at *m*/*z* 463 and 317, due to the losses of 152 Da and 146 Da, which were indicative of a galloyl (gallic acid−H_2_O) and a deoxyhexose moiety, respectively. The peak at *m*/*z* 169 in their MS/MS spectra also implied the presence of the galloyl group. Analogously, **126** and **128** were assumed to be quercetin-3-*O*-galloyl-deoxyhexoside isomers as they showed their [M−H]^−^ ion at *m*/*z* 599 and a fragment ion at *m*/*z* 301 corresponding to the loss of a galloyl-deoxyhexose unit (−298 Da). Similarly, **123** was identified as kaempferol-3-*O*-galloyl-hexoside [66,67,76]. 

Compounds **87**, **114**, **112**, and **99** were found to be methoxylated flavones. Neutral losses of 15 Da referred to the cleavage of methyl radicals (−CH_3_^•^) indicating the presence of methoxy groups in the molecule; thus, **87**, **114**, **112**, and **99** were identified as trihydroxy-dimethoxyflavone, hexahydroxy-methoxyflavone, tetramethoxyflavone-*O*-deoxyhexoside, and a tetramethoxyflavone derivate [59]. The dihydroxy-methoxyflavanone-*O*-hexoside (**130**) presenting the [M−H]^−^ ion at *m*/*z* 447 was identified based on its [M−H−162]^−^ ion at *m*/*z* 285 [70].

#### 2.1.2. Characterization and Analysis of Catechin Derivatives

Catechin and epicatechin derivates exhibited a typical fragment ion in their mass spectra at *m*/*z* 289 during their mass spectrometric fragmentation. Compound **93** also presented this fragment which arose from the loss of a 152 Da galloyl moiety (*m*/*z* 441 → 289); therefore, **93** was characterized as catechin-gallate or epicatechin-gallate [60]. The [M−H]^−^ ion of the catechin dimer **21** showed a mass-to-charge ratio of *m*/*z* 575, 2 Da less than the B-type procyanidin dimers, due to the additional C-O-C linkage that occurs in A-type procyanidins. Hence, compound **21** was identified as an A-type procyanidin dimer [45,60]. 

#### 2.1.3. Characterization and Analysis of Gallic Acid Derivates

Gallic acid derivatives presented a diagnostic fragment ion at *m*/*z* 169 corresponding to the deprotonated molecular ion of gallic acid. An additional fragment ion at *m*/*z* 125 was also formed by the cleavage of the carboxyl group from gallic acid [41]. In gallic acid derivates conjugated with sugars, the loss of 162 Da denotes a hexose moiety and the loss of 132 Da a pentose moiety. Therefore the [M−H]^−^ ion at *m*/*z* 331 indicates a monogalloyl-hexose isomer (**3**, **4**, **5**, **7**, **10**, **11**, **12**, **15**, **35**), and the [M−H]^−^ ion at *m*/*z* 301 corresponds to galloyl-pentose (**34**) [38]. 

Compounds **6** and **8** produced [M−H]^−^ ion at *m*/*z* 481 and generated fragment ions at *m*/*z* 301 and 275, corresponding to an HHDP residue and a decarboxylated HHDP moiety, respectively. The loss of 180 Da indicated a hexose; therefore, compounds **6** and **8** were identified as mono-HHDP-hexose isomers. Similarly, compounds **22**, **26**, **28**, and **38** at *m*/*z* 783 were identified as bis-HHDP-hexose isomers, showing fragment ions in their MS/MS spectra at *m*/*z* 481, 301 and 275, corresponding to HHDP-hexose, the HHDP residue, and the decarboxylated HHDP moiety, respectively [42,46,47]. 

Compounds **25**, **31**, **44,** and **56** were identified as galloyl-HHDP-hexose due to their deprotonated molecular ions [M−H]^−^ at *m*/*z* 633 and fragment ions at *m*/*z* 481 (loss of a galloyl moiety [M−H−152]^−^, HHDP-hexoside), *m*/*z* 463 (loss of a gallic acid [M−H−170]^−^), and *m*/*z* 301 (loss of a galloyl hexose [M−H−152−180]^−^ [46,48,52]. Similarly, compounds **81**, **91**, and **97** with an [M−H]^−^ ion at *m*/*z* 937 were tentatively identified as trigalloyl-HHDP-hexoside isomers [48,58]. Compounds **32**, **36**, **39**, **42**, and **96,** presenting a pseudomolecular ion at *m*/*z* 951, were galloyl-HHDP-DHHDP-hexose isomers [38,41,48].

Gallotannins consist of a polyol (usually a glucose) core which is esterified through its hydroxyl groups by galloyl units to form polymers. Gallotannins also present the aforementioned fragment ions of gallic acid at *m*/*z* 169 and 125 as well as typical neutral losses of 170, 152, and 134 Da equaling to gallic acid, a galloyl moiety, and a galloyl moiety losing a water molecule, respectively. Trigalloyl-hexose isomers (**40**, **48**, **54**, **57**, **61**, **62**, **66**) were detected displaying the [M−H]^−^ ion at *m*/*z* 635. The fragment ions were formed by the cleavage of gallic acid [M−H−170]^−^ at *m*/*z* 465, and by the loss of a galloyl moiety [M−H-170−152]^−^ at *m*/*z* 313 as well. Tetragalloyl-hexose (**78**, **85**, **89**, **101**) and pentagalloyl-hexose isomers (**94**, **98**, **102**, **105**, **113**, **122**) exhibited [M−H]^−^ ions at *m*/*z* 787 and 939, respectively [38,41]. 

Methylgallic acid (**53**) was identified due to its [M−H]^−^ ion at *m*/*z* 183. Besides this methylgallic acid anion fragment ion, methylgalloyl-*O*-hexose (**23**) showed [M−H]^−^ ion at *m*/*z* 354. Compounds **69**, **107**, and **111** exhibited their [M−H]^−^ ions at *m*/*z* 335 and fragment ions at *m*/*z* 183 generated by the loss of 152 Da that corresponds to a galloyl moiety. Therefore, **69**, **107**, and **111** were characterized as galloyl-methylgallic acid isomers [42]. 

Compound **88** produced a [M−H]^−^ ion at *m*/*z* 463 (ellagic acid+162 Da) and a prominent fragment ion at *m*/*z* 301 (ellagic acid residue); therefore, it was presumed to be ellagic acid-*O*-hexoside. Compound **95** was a galloyl-quinic acid derivate. The fragment ion at *m*/*z* 343 in its MS/MS spectrum implied the presence of a gallic acid moiety linked to a dehydrated quinic acid (169 + 174 Da) [38]. 

#### 2.1.4. Characterization and Analysis of Naphthoquinones and Tetralones

Typical components of the *J. nigra* samples were naphthoquinones. Continuous cleavage of H_2_O (18 Da) and CO (28 Da) was diagnostic of their fragmentation. Dihydroxy-naphthoquinone (**9**) was characterized due to its [M−H]^−^ ion at *m*/*z* 189 [43]. Trihydroxy-tetralone (**83**) presented the pseudomolecular ion at *m*/*z* 193 and a fragment at *m*/*z* 175 [M−H−H_2_O]^−^ [53]. Compounds **50** and **52** were tentatively identified as trihydroxy-tetralone-*O*-hexoside isomers, due to their aglycone anion fragment at *m*/*z* 193 in the MS/MS spectrum, which was formed by the loss of a 162 Da (hexose moiety) [53,54]. After its isolation and NMR analysis, an additional trihydroxy-tetralone derivative (**92**) was identified as 1,2,3,4-tetrahydro-7,8-dihydroxy-4-oxonaphthalen-1-yl-6-*O*-galloyl-glucoside [53]. 

Hydrojuglone-*O*-hexoside (**75**) yielded fragment ions at *m*/*z* 175 and 131, due to the loss of a hexose (*m*/*z* 337 → 175) and a carboxyl (*m*/*z* 175 → 131) moiety, respectively [31,53,57]. Hydrojuglone (**129**) was also detected in the extracts as an abundant constituent. The main component of the chloroform extract was juglone (**141**) [38,40,54,55]. Compounds **156** and **159** were presumed to be bisjuglone isomers as they displayed [M−H]^−^ ion at *m*/*z* 345 and fragment ions at *m*/*z* 317 [M−H−CO]^−^, 301 [M−H−CO_2_]^−^, 289 [M−H−2CO]^−^, and 261 [M−H−3CO]^−^ [55,57]. Similarly, **161** was tentatively identified as trisjuglone based on its [M−H]^−^ ion at *m*/*z* 515. 

#### 2.1.5. Characterization and Analysis of Caffeic Acid Derivatives

Compound **1** producing the deprotonated molecular ion at *m*/*z* 341 and a characteristic product ion of caffeic-acid [M−H−162]^−^ was identified as caffeoyl-*O*-hexose. Compounds **30** and **47** as well as **41** presented [M−H]^−^ ions at *m*/*z* 353 and 337, respectively; therefore, these were identified as caffeoyl- and coumaroylquinic acids. The position of the caffeoyl or coumaroyl moiety could also be proposed based on the relative intensities of the fragment ions. In case of **30** and **41**, the abundance of the *m*/*z* 191 (deprotonated quinic acid) fragment ion and the *m*/*z* 179 (deprotonated caffeic acid) or the *m*/*z* 163 (deprotonated ferulic acid) fragment ions referred to 3-*O*-caffeoylquinic acid and 3-*O*-coumaroylquinic acid, respectively. Compound **47**, presenting the abundant base peak at *m*/*z* 191 with very high relative intensity, was identified as 5-*O*-caffeoylquinic acid [49,50].

#### 2.1.6. Characterization and Analysis of Benzoic Acid and Other Organic Derivatives 

Dihydroxybenzoyl-*O*-hexoside (**20**) was detected displaying a parent ion at *m*/*z* 315 as well as a fragment ion at *m*/*z* 153, corresponding to the loss of a hexose moiety and a dihydroxybenzoic acid aglycone [38,41]. Based on the literature data, **33, 37**, and **45** were tentatively identified as hydroxyl-dimethoxybenzoyl-*O*-hexoside, presumably syringic acid-*O*-hexoside. The [M−H]^−^ ion at *m*/*z* 359 and the fragment ions at *m*/*z* 197 and 182 indicated the loss of a hexose (162 Da) and a methyl (15 Da) moiety, respectively [38,41]. Syringic acid has already been reported for black walnut [22,37]. An organic acid, malic acid (**2**) was recognized due to its [M−H]^−^ ion at *m*/*z* 133 and the fragment ion [M−H−H_2_O]^−^ at *m*/*z* 115 [39,40]. Peaks **135** and **139** presenting their [M−H]^−^ ions at *m*/*z* 327 and 329 were tentatively identified as oxo-dihydroxy-octadecenoic acid and trihydroxy-octadecenoic acid, respectively [72]. 

#### 2.1.7. Characterization and Analysis of Diarylheptanoids

Diarylheptanoids have already been reported for other Juglans species [4,5]; however, they have not been detected in *J. nigra* yet. Based on this, we presumed their presence in our samples. Some glycosidic derivatives have been detected, where the neutral loss of 180 Da referred to a hexose residue (e.g., **18**), while that of 150 Da pointed to a pentose moiety (e.g., **63**, **119**) [16]. The linear diarylheptanoids **18, 63**, and **119** presenting the [M−H]^−^ ions at *m*/*z* 493 and 463, respectively, were identified due to their typical fragment ions at *m*/*z* 331 and 313 [16,38,77]. The cyclic diarylheptanoid **140** presented the [M−H]^−^ ion at *m*/*z* 341 and the fragments *m*/*z* 297 [M−H−CO_2_]^−^, and *m*/*z* 269 [M−H−CO_2_]^−^. Compound **145** ([M−H]^−^ at *m*/*z* 327) yielded fragment ions at *m*/*z* 312 [M−H−CH_3_]^−^, *m*/*z* 253 [M−H−C_4_H_10_O]^−^, and *m*/*z* 239 [M−H−C_5_H_12_O]^−^. Therefore, **140** and **145** were tentatively identified as juglanin G and B, respectively [38,43,45,55,78]. Compounds **134**, **138**, and **151** were presumed to be additional cyclic diarylheptanoid aglycones.

### 2.2. Structural Characterization of the Isolated Compounds 

**Figure 2:**
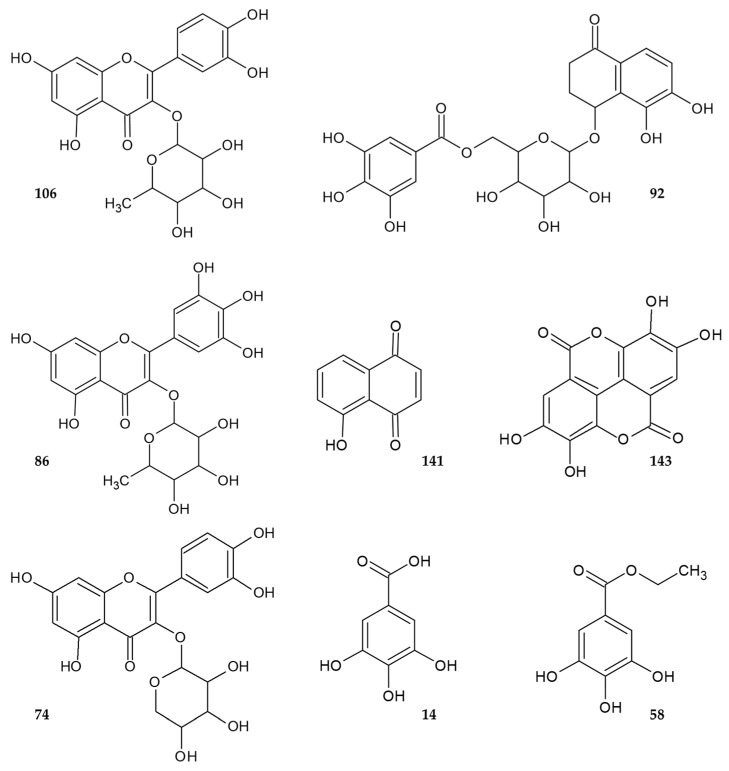
Compounds isolated from the pericarp of *J. nigra*.

In order to unambiguously identify their structures, three flavonol-*O*-glycosides (**74**, **86**, **106**), one tetralone-glycoside (**92**), one naphthoquinone (**141**), two gallic acid derivatives (**14**, **58**), and ellagic acid (**143**) were isolated by C_18_ flash chromatography followed by multiple successive C_18_ (semi-)preparative HPLC separations. The structures of the compounds were elucidated by HR-ESI-MS analyses, by comparing their retention times and mass spectrometric fragmentation with those of standard substances. Additional 1D and 2D NMR experiments were performed in the case of compound **92**. Figure 2 presents the structures of the isolated constituents and Table 2 summarizes their high-resolution mass spectrometric data. 

Based on our LC-MS analyses, we proposed a trihydroxy-tetralone skeleton with an attached galloyl-hexose moiety for compound **92**. The assumed structure was proven by NMR spectroscopy. ^1^H and ^13^C spectrum were in good agreement with the literature data [79]. In the aromatic region of the ^1^H NMR spectrum, three distinct signals were identified. The singlet at 6.99 ppm representing two hydrogens was attributed to the two aromatic protons of gallic acid, while the two coupled doublets at 7.15 ppm and 6.81 ppm representing 1-1 protons were identified as the aromatic protons of the 1,2,3,4-tetrahydro-7,8-dihydroxy-4-oxonaphthalen substructure. In the aliphatic part of the spectrum, the signals of a β-glucopyranose ring were identified, along with two pairs of methylene protons and the methine proton of the naphthalene derivative between 3.0 and 2.0 ppm and at 5.22 ppm, respectively. The linkage of gallic acid to the C-6 position of the glucopyranose ring was proved by three-bond correlation between the carbonyl carbon of gallic acid and the H-6 of glucose. Similarly, an intensive three-bond correlation was identified between H-1 of glucose and C-1 of the naphthalene moiety. The indicative signal of the ketone was also found in the ^13^C spectrum at 206.1 ppm. Accordingly, **92** was identified as 1,2,3,4-tetrahydro-7,8-dihydroxy-4-oxonaphthalen-1-yl-6-*O*-[(3,4,5-trihydroxyphenyl)carbonyl]-β-D-glucopyranoside. Atom numbering for the chemical shift assignment as well as ^1^H and ^13^C NMR spectra of compound **92** are presented in Supplementary Figures S4–S6.

### 2.3. Determination of the In Vitro Antiproliferative Activity 

For the evaluation of the in vitro antiproliferative activity of the compounds, the cell viability was determined by resazurin (Alamar Blue) assay on MDA-MB 231 breast carcinoma, A2058 melanoma, and HT-29 colon carcinoma cell culture. The control wells were treated only with serum-free medium. The IC_50_ is the micromolar (μM) compound concentration required for 50% inhibition of the cells’ viability carefully calculated from dose–response curves (Table 3). 

Compounds **141** and **14** induce cytostasis on MDA-MB 231 cells with IC_50_ of 9.9 and 49.8 µM, respectively. The other compounds have no in vitro effect on these cells. On the melanoma culture, compound **141** and compound **14** showed a fair antiproliferative effect (IC_50_ = 15.5 and 57.2 µM). Other compounds had no or limited inhibiting activity. Compounds **143**, **14**, and **141** revealed IC_50_ values of 12.1, 0.53, and 71.2 µM, respectively, on HT-29 cell culture. 

To determine the selectivity, we assessed the compounds on the Vero E6 non-tumorous kidney cells from African green monkeys (*C. sabaeus*), and their cytostatic activity was determined (Table 3). Compounds **106, 86**, **141**, **92**, **14**, and **58** showed cytostatic activity on the Vero E6 cells with IC_50_ values of 15,9, 3.7, 3.7, 61.1, 49.9, and 82.1 µM, respectively. Compounds **143** and **74** were not cytostatic on the Vero E6 cells. 

We also determined the IC_50_ value for daunomycin and tamoxifen, which are highly active, as suggested by the IC_50_ values value, but very toxic and not selective compounds. As positive controls, we applied daunomycin and tamoxifen as clinically used reference compounds. Daunomycin (isolated from *Streptomyces peucetius* [80]) is among the first cytostatic compounds and the most employed, active on different tumors (e.g., leukemia, lymphoma, breast, and lung cancers) as an anthracycline aminoglycoside agent. A 50% inhibition in all cell lines is demonstrated, reported here at 0.2–1.1 μM concentration (IC_50_ value). Daunomycin is a DNA intercalating molecule [81] and is not a selective compound with many side effects (partly due to its low IC_50_ value). Tamoxifen treats estrogen-receptor-positive breast cancers as well as preventing the incidence of breast cancer in high-risk populations. Structural analysis showed tamoxifen is a non-steroidal antiestrogen used, and its metabolites bind DNA via hydrophobic and hydrophilic interactions [82].

### 2.4. Parallel Artificial Membrane Permeability Assay (PAMPA)

Among the compounds investigated, only juglone (**141**) had a log*P_e_* value greater than −5.0 (−4.41 ± 0.10) in the PAMPA-GI experiments; meanwhile, in the PAMPA-BBB studies, juglone (**141**) and ethyl gallate (**58**) possessed log*P_e_* values greater than −6.0 (−4.11 ± 0.19 and −5.77 ± 0.31, respectively) (Table 4). 

Accordingly, these constituents can be considered to have good membrane penetration ability [83]; thus, they can be expected to be absorbed in the gastrointestinal tract and cross the blood–brain barrier by transcellular passive diffusion. However, it should be noted that, for both juglone (**141**) and ethyl gallate (**58**), additional minor compounds (comprising <10% in the case of juglone (**141**) and <5% for ethyl gallate (**58**) based on UHPLC-DAD) appeared in the aqueous solutions prepared for the PAMPA experiments, indicating that some kind of chemical reactions had occurred. These are most likely to be the reduction of juglone (**141**) to hydrojuglone and the ester hydrolysis reaction of ethyl gallate (**58**). Although such small changes in compound concentration are not expected to significantly affect the results, given the accuracy of the PAMPA, they should be treated with caution.

Compounds **14**, **74 86**, **92**, and **106** were not detected in the acceptor phase of the PAMPA models; meanwhile, the calculated log*P_e_* value for ellagic acid (**143**) was less than −6.0 in both assays. This indicates that these compounds are unable to traverse the lipid membranes of the gastrointestinal tract (GIT) and the blood–brain barrier (BBB) via passive diffusion. Among these, myricetin-3-*O*-rhamnoside (**86**) and quercetin-3-*O*-xyloside (**74**) showed significant degree of degradation in the pH 7.4 buffer after 4 h of incubation at 37 °C [84]. Nonetheless, the negative results of the PAMPA-BBB experiments can be considered valid, as both compounds are unlikely to cross the model membranes via passive diffusion due to their high molar mass (>430 g/mol) and polarity (*c*log*P* < 1).

## 3. Discussion

We have performed a comprehensive phytochemical screening of *J. nigra* leaf, bark, and pericarp samples. In the present study, we tentatively characterized 161 phenolic compounds in *J. nigra* extracts by UHPLC-MS/MS. In line with the literature data, we detected flavonoids, phenolcarboxylic acids, ellagitannins, and naphthoquinones. Additionally, we described several gallotannins, flavonoids, juglone and tetralone derivatives, and linear and cyclic diarylheptanoids in *J. nigra* for the first time. We observed prominent differences between the composition of the different plant parts. Flavonol glycosides were dominant in the leaf and bark extracts, flavanone and chalcone glycosides occurred only in the bark, while gallotannins and ellagitannins prevailed in pericarp samples. Naphthoquinones and tetralones were present in all parts of the plant. The most prevalent constituents of the samples were hydrojuglone-*O*-hexoside (**75**), myricitrin (**86**), and quercitrin (**106**). 

Glycosides of the flavonols quercetin and myricetin have been identified in the nuts previously [22,24,32,37]. Quercitrin and myricitrin have also been isolated from the leaves, while the latter was found in the bark, too [26,27,31]. In accordance with these, we confirmed quercetin-3-*O*-rhamnoside (quercitrin, **106**) and myricetin-3-*O*-rhamnoside (myricitrin, **86**) in bark, leaf, and pericarp samples. Additionally, we characterized quercetin-3-*O*-hexosyl-deoxyhexoside (**80**), quercetin-3-*O*-xyloside (**74**), quercetin-3-*O*-hexoside (**90**), and myricetin-3-*O*-hexoside (**77**) in those plant parts for the first time. We also reported the presence of myricetin-3-*O*-galloyl-deoxyhexoside isomers (**116** and **117**) as well as quercetin-3-*O*-galloyl-deoxyhexoside isomers (**126** and **128**), which have been detected in black walnut for the first time. These flavonol acylglycosides were characteristic of all samples.

Flavanone and chalcone glycosides sakuranin and neosakuranin have been isolated from black walnut [27,31]. Based on this, we tentatively identified the following compounds: trihydroxy-methoxychalcone-*O*-hexoside (presumably neosakuranin, **109**), dihydroxy-methoxyflavanone-*O*-hexoside (supposedly sakuranin, **130**), and additional di- and monoglycosides of dihydroxy-, trihydroxy-, and tetrahydroxy-methoxyflavanones (**100**, **131**, and **121**, respectively). 

Lin et al. evaluated bioactive ellagitannins, gallotannins, and catechin derivatives as well as gallic acid and ellagic acid from the kernels [20,22,37]. Several digalloyl-hexahydroxydiphenoyl-hexose (digalloyl-HHDP-hexose, presumably tellimagrandin I) isomers (**43**, **49**, **55**, **60**, **68**) as well as bis-HHDP-hexose (supposedly pedunculagin) isomers (**22**, **26**, **28**, **38**) were also present in our extracts. However, we did not detect any punicalin isomers. In line with the literature, trigalloyl-hexose (**40**, **48**, **54**, **57**, **61**, **62**, **66**) and pentagalloyl-hexose (**94**, **98**, **102**, **105**, **113**, **122**) isomers were also detected. Nevertheless, other gallotannins have not been previously reported for black walnut. We revealed several gallo- and ellagitannin derivatives in *J. nigra* for the first time: tetragalloyl-hexoses (**78**, **85**, **89**, **101**), trigalloyl-HHDP-hexoses (**81**, **91**, **97**), galloyl-bis-HHDP-hexoses (**73**, **76**, **79**, **82**, **84**), a galloyl-hexahydroxydiphenoyl-dehydrohexahydroxydiphenoyl-hexose (galloyl-HHDP-DHHDP-hexose, **96**), and an ellagitannin (castalagin) derivative (**72**). We also reported gallocatechin-*O*-gallate or epigallocatechin-*O*-gallate (**67**) and an A-type procyanidin dimer (**21**) in black walnut for the first time. 

Tetralone derivatives and naphthoquinones are also representative constituents of *J. nigra* and other *Juglans* species [29,30]. Gupta et al. isolated hydrojuglone-*O*-glucoside from the bark of *J. nigra* [31] and we also identified it (**75**) as the most predominant naphthalene derivative in our samples. Additionally, we observed hydrojuglone (**129**), two bisjuglone isomers (**156**, **169**), and trisjuglone (**161**), as well as trihydroxy-tetralone (**83**), two trihydroxy-tetralone-*O*-hexoside isomers (**50**, **52**), and trihydroxy-tetralone-*O*-galloyl-glucoside (**92**), which have not been previously described for black walnut.

Although some of its constituents, characteristically the naphthoquinones and phenolic acid derivatives, have been known to exert cytotoxic actions [8,85,86,87], biological activities and pharmacokinetic properties of black walnut polyphenols have not been characterized yet. Therefore, we have also performed an in vitro antiproliferative assay with a representative compound set in human cancer cell cultures. Juglone (**141**) demonstrated similar cytostatic activity on MDA-MB 231 and A2058 cells as the clinical standards. Gallic acid (**14**) was one order of magnitude less effective but had moderate selectivity. Further research is needed to investigate the mechanism of action regarding the promising new cytostatic candidates. It is important to note that their effect is in a similar concentration range as the natural compound vermelhotin (from fungal endophytes origin), which causes 50% inhibition at 12–20 µM concentrations depending on cell culture [88].

As shown by our previous results, only a handful of natural products may be able to cross biological membranes of the gastrointestinal tract or the blood–brain barrier via passive diffusion in the PAMPA experiments [84,89]. In this study, we demonstrated that most of the major constituents of black walnut pericarp cannot be considered to have satisfactory membrane penetration ability. Based on the results of the PAMPA experiments, only juglone (**141**) and ethyl gallate (**58**) are able to cross the membranes of the GIT and the BBB by passive diffusion. 

Juglone (**141**) showed both good membrane permeability and cytostatic activity. Furthermore, based on our PAMPA-BBB results, it can be expected to reach the central nervous system (CNS). Therefore, as suggested by other studies as well [90,91], it may be a suitable candidate for future research into antitumor agents targeting the CNS.

Gallic acid (**14**) exhibited moderate but not selective cytostatic activity and poor membrane permeability in the PAMPA model. This latter result is in accordance with those of previous studies [92,93], which indicated that it is moderately absorbed in the gastrointestinal tract via paracellular transport. Based on our results and the net negative surface charge of endothelial cells in the blood–brain barrier, refusing to accept negatively charged compounds [94], transcellular passive diffusion of gallic acid (**14**) across the BBB is very unlikely. The results taken together suggest that gallic acid (**14**), in contrast to juglone (**141**), is not an ideal candidate for research into the therapy of central nervous system tumors. However, given its moderate absorption rate and cytostatic activity on the HT-29 colon carcinoma cell line, further studies in this direction would be worthwhile.

Ellagic acid (**143**) exerted moderate and selective cytostatic effect on the HT-29 colon cancer cell line. Considering this, the poor–moderate transcellular membrane penetration ability of compound **143** in the PAMPA-GI model, which is also in agreement with the literature data [85], may even be beneficial. The remaining isolated compounds (**86, 92, 106, 74**) had no effect on the tumor cell lines, nor did they show good permeability in the PAMPA experiments. 

In vitro antioxidant, anti-inflammatory, and anticancer activities of black walnut and its constituents have been shown. However, in a human study, consumption of black walnuts did not cause any significant difference in LDL antioxidant capacity [24]. Several factors might play a role in this negative result, with the possible inability of the antioxidative constituents to be absorbed in the gastrointestinal tract being one. On the other hand, black walnut bark extract and juglone showed in vivo neuroprotective effects in cerebral ischemia in rats [35]. 

According to our results, compounds showing the ability to cross biological membranes (e.g., **141**, **58**) and/or antiproliferative activity (e.g., **141**, **14**) presumably contribute to the biological activities of the *J. nigra* extracts. However, the role of the other components cannot be neglected, as they may be converted in the human body to metabolites with better bioavailability. Furthermore, due to the artificial nature of the membrane used in the PAMPA, only passive transport mechanisms can occur, and active transport cannot be studied. It should be noted that results might also be influenced by the accuracy of the analytical method that was applied to quantify the compounds. In some cases (e.g., for **14** and **74**), inter-day accuracy was outside ±20% at the lowest concentration level, partly due to phenolic compounds often being susceptible to decomposition. Therefore, further studies aimed at investigating the biological activity and pharmacokinetic properties of *J. nigra* constituents would be of great interest. 

## 4. Materials and Methods

### 4.1. Solvents and Chemicals

Chloroform, ethyl acetate, and methanol of reagent grade as well as HPLC-grade methanol and acetonitrile were purchased from Molar Chemicals Kft. (Halásztelek, Hungary). The HPLC-grade acetic acid and formic acid were delivered by Sigma-Aldrich (Budapest, Hungary). High-purity water was gained by a Millipore Direct Q5 Water Purification System (Billerica, MA, USA).

Dimethyl sulfoxide (DMSO), *n*-dodecane, hydrochloric acid (HCl), sodium hydroxide (NaOH), disodium hydrogen phosphate heptahydrate (Na_2_HPO_4_ · 7H_2_O), and sodium dihydrogen phosphate monohydrate (NaH_2_PO_4_ · H_2_O) were obtained from Reanal-Ker (Budapest, Hungary), while caffeine and rutin standards, phosphatidylcholine, cholesterol, the porcine polar brain lipid extract, and the PBS tablet (phosphate-buffered saline, pH 7.4) were purchased from Merck (Darmstadt, Germany).

### 4.2. Plant Material and Sample Preparation

Pericarp samples of *Juglans nigra* were collected in Hungary, in the Fiumei Road Cemetery (Budapest, October 2021); later, the leaves and bark were collected at the same place (Budapest, June 2022). Authenticated samples and herbarium specimens are deposited at the Herbarium of the Department of Pharmacognosy, Semmelweis University, Budapest, Hungary. 

For the analytical studies, the dried and milled samples (1 g) were extracted in an ultrasonic bath (Bandelin Sonorex Digitec DT 1028, Berlin, Germany) with chloroform, ethyl acetate, and methanol, consecutively (3 × 20 mL for all solvents, 30 min each), at room temperature. The extracts were distilled to dryness with a rotary evaporator (Büchi Rotavapor R-200, Flawil, Switzerland) at 40 °C. The samples were suspended in HPLC-grade methanol and filtered through Phenomenex RC 15 mm 0.2 µm syringe filters (Torrance, CA, USA). For the isolation of the main constituents, the pericarp samples (649 g) were prepared in the same way, except for the volume of the solvents (2 L) and the extraction time (2 h).

### 4.3. Isolation of Compounds from J. nigra Pericarp

For the isolation of the main constituents, *J. nigra* pericarp sample was collected in the Fiumei Road Cemetery (Budapest, October 2021). Similarly to the analytical samples, the lyophilized pericarp was extracted in an ultrasonic bath with chloroform, ethyl acetate, and methanol successively (3 × 2 L for all solvents, 2 h each).

The chloroform extract was evaporated to dryness under reduced pressure at 40 °C and suspended in methanol (final concentration: 0.1 g/mL). The extract was then fractionated by flash chromatography (CombiFlash NextGen 300+, Teledyne Isco, Lincoln, NE, USA), using a RediSep Rf Gold Silica gel column (40 g, Teledyne Isco) as stationary phase. Eluent A was acetone, eluent B was *n*-hexane. The following gradient elution was applied: 0% B (0.0–3.0 min), 0–100% B (3.0–43.0 min), 100% B (43.0–60.0 min), and a flow rate of 40 mL/min. The separation process was repeated two more times to use up all the extract. Fractions of 16 mL each were collected and fractionated further by semi-preparative and preparative HPLC.

From the first flash separation, fractions 16–21 were combined with fractions 19–21 from the second chromatography and fractions 20–21 from the third run. The combined fractions were chromatographed again on a RediSep Rf Gold Silica gel column (24 g, Teledyne Isco). The conditions were as follows: isocratic elution (*n*-hexane:acetone:formic acid; 18.5:1.5:0.2 *v*/*v*/*v*); a flow rate of 35 mL/min; a run time of 60.0 min. Fraction 7 was diluted in dichloromethane and extracted with 3 × 10 mL distilled water. The organic phase was then further separated by preparative HPLC (Hanbon Sci. & Tech. Newstyle, Huaian, Jiangsu, China), using a Gemini NX-C18 (150 × 21.2 mm, 5 μm; Phenomenex Inc.) column and isocratic elution (25% eluent A: methanol, 75% eluent B: formic acid in water (0.1%), flow rate: 12 mL/min) to obtain **141** (t_R_ = 26.0 min).

The ethyl acetate extract of the pericarp was evaporated to dryness under reduced pressure at 40 °C and suspended in methanol (final concentration: 0.1 g/mL). The extract was then fractionated by flash chromatography using the same instrumentation and a RediSep Rf Gold C18 column (100 g, Teledyne Isco) as stationary phase. Eluent A was 0.3% acetic acid in water, eluent B was methanol (gradient elution: 5% B 0.0–3.0 min, 5–20% B 3.0–4.0 min, 20–100% B 4.0–34.0 min, 100% B 34.0–50.0 min; flow rate: 60 mL/min). Fractions of 16 mL each were collected and further fractionated by semi-preparative HPLC.

Combined fractions 13–32 were separated by semi-preparative HPLC (Waters 2690 HPLC system, Waters Corporation, Milford, MA, USA). The Luna C18 100 A (150 × 10 mm i.d., 5 μm; Phenomenex) column as stationary phase, and 0.3% acetic acid in water (as eluent A), and methanol (as eluent B) were used. The following gradient elution was applied to obtain **14** (t_R_ = 9.0 min): 0.0–10.0 min 35–65% B, 10.0–11.0 min 65–100% B, 11.0–16.0 min 100% B (flow rate: 1 mL/min).

Fractions 45–50 from the ethyl acetate extract were combined and further chromatographed by preparative HPLC (Hanbon System) using a Gemini NX-C18 (150 × 21.2 mm, 5 μm; Phenomenex Inc.) column as stationary phase to collect 6 subfractions. The following gradient elution was used (eluent A: 0,1% formic acid in water, eluent B: methanol, flow rate 12 mL/min): 0.0–30.0 min: 15–20% B; 30.0–31.0 min: 20–100% B; 31.0–38.0 min: 100% B. Subfraction 2 was separated using the same Waters 2690 HPLC instrument and Luna C18 (150 × 10 mm i.d., 5 μm; Phenomenex Inc.) column. Isocratic elution was applied at 23% B where eluent A was 0.3% acetic acid in water, eluent B was acetonitrile, the flow rate of the mobile phase was 2.1 mL/min, and the separation yielded **58** (t_R_ = 9.5 min).

Half the quantity of the combined fractions 57–61 from the ethyl acetate extract were separated by semi-preparative HPLC (Waters 2690 HPLC system), and the stationary phase was a Luna C18 (150 × 10 mm i.d., 5 μm; Phenomenex Inc.) column. Eluent A was 0.3% acetic acid in water and eluent B was methanol, gradient elution: 0.0–10.0 min 50–60% B, 10.0–11.0 min: 60–100% B, 11.0–16.0 min: 100% B. We obtained **86** (t_R_ = 12.5 min) and a subfraction, which was further separated with the same instrumentation, column, and eluents but this time using isocratic elution (0.0–12.0 min: 55% B) at a flowrate of 1.0 mL/min that resulted in **92** (t_R_ = 9.5 min). The other half of the combined fractions 57–61 was first fractioned by the previously mentioned preparative HPLC and Gemini NX-C18 column (150 × 21.2 mm, 5 μm; Phenomenex Inc.). Then, **92** (t_R_ = 8.6 min) was isolated from fraction 5a using isocratic elution: 0.0–12.0 min: 55% B at a flowrate of 1.2 mL/min (eluent A: 0.3% acetic acid in water, eluent B: methanol). Subfraction 5b was further separated into **92** (t_R_ = 8.6 min) and **86** (t_R_ = 9.3 min) with the same Waters 2690 HPLC instrument and Luna C18 (150 × 10 mm i.d., 5 μm; Phenomenex Inc.) column, with 0.3% acetic acid in water (A) and methanol (B) as eluents. Isocratic elution was used with 55% eluent B, the flowrate was 1.2 mL/min.

Combined fractions 62–69 from the ethyl acetate extract were chromatographed with the same Waters 2690 HPLC instrument and Luna C18 (150 × 10 mm i.d., 5 μm; Phenomenex Inc.) column as stationary phase. Eluent A was 0.3% acetic acid in water, eluent B was methanol, flow rate of the mobile phase was 1 mL/min. Utilizing the following gradient elution (0.0–10.0 min 55–60% B, 10.0–11.0 min 60–100% B, 11.0–16.0 min 100% B), we obtained **106** (t_R_ = 13.7 min).

Fractions 119–130 from the ethyl acetate extract were united and further separated with the previously described device (Waters 2690 HPLC), column Luna C18 (150 × 10 mm i.d., 5 μm; Phenomenex Inc.), and eluents (eluent A: 0.3% acetic acid in water, eluent B: methanol). The applied gradient was as follows: 0.0–20.0 min: 80–100% B, 20.0–30.0 min: 100% B. The separation resulted in **143** (t_R_ = 8.0 min).

The methanol extract of black walnut pericarp was evaporated to dryness under reduced pressure at 40 °C and suspended in methanol. The extract was then fractionated by flash chromatography (CombiFlash NextGen 300+), using a RediSep Rf Gold C18 column (100 g, Teledyne Isco) as stationary phase. Eluent A was 0.3% acetic acid in water and eluent B was methanol (gradient elution: 5% B (0.0–4.0 min), 5–20% B (4.0–5.0 min) 20–65% B (5.0–23.0 min), 65–100% B (23.0–30.0 min), 100% B (30.0–40.0 min); flow rate: 60 mL/min). Fractions of 16 mL each were collected and further fractionated by (semi-)preparative HPLC.

The combined fractions 106–115 were separated by preparative HPLC using the Gemini NX-C18 (150 × 21.2 mm, 5 μm; Phenomenex Inc.) column as stationary phase. A gradient elution was applied: 0.0–30.0 min 17–22%, 30.0–30.5 min 22.0–100.0% B, 30.5–34.0 min 100% (eluent A was 0.1% formic acid in water, eluent B was acetonitrile) to obtain **143** (t_R_ = 20.5 min).

Another charge of the methanol extract was also prepared as described before, using 157 g of the same *J. nigra* dried sample and 3 × 800 mL methanol. The extract was evaporated to dryness under reduced pressure at 40 °C. A measure of 7.5 g of the dried extract was adsorbed to silica gel and chromatographed with flash chromatography (CombiFlash NextGen 300+), using a RediSep Rf Gold C18 column (150 g, Teledyne Isco). Eluent A was 0.1% formic acid in water and eluent B was methanol. The following gradient elution was applied: 10% B (0.0–2.0 min), 10–50% B (2.0–32.0 min), 50–100% B (32.0–42.0 min); flow rate of 85 mL/min. Fractions 111–122 were collected and separated by semi-preparative HPLC (Waters 2690 HPLC system). The conditions were as follows: stationary phase: Luna C18 (150 × 10 mm i.d., 5 μm; Phenomenex Inc.) column, isocratic elution: formic acid in water (0.1%) (A), acetonitrile (B); 81:19, *v*/*v*; flow rate: 1.7 mL/min. The chromatography resulted in **74** (t_R_ = 10.0 min) and **92** (t_R_ = 21.0 min).

The quantity of the isolated substances was as follows: gallic acid (**14**) (1.1 mg), ethy gallic acid (**58**) (2,5 mg), myricetin-3-*O*-rhamnoside (**86**) (4.6 mg), trihydroxy-tetralone-*O*-galloyl-hexoside (**92**) (16.5 mg), quercetin-3-*O*-rhamnoside (**106**) (1.0 mg), ellagic acid (**143**) (4.8 mg), juglone (**141**) (13.7 mg), and quercetin-3-*O*-xyloside (**74**) (5 mg).

**1,2,3,4-tetrahydro-7,8-dihydroxy-4-oxonaphthalen-1-yl-6-*O*-[(3,4,5-trihydroxyphenyl)carbonyl]-β-D-glucopyranoside (92)**

^1^H NMR (400 MHz, DMSO-*d*_6_) δ 7.15 (d, *J* = 9.0 Hz, 1H), 6.98 (s, 2H), 6.81 (d, *J* = 9.0 Hz, 1H), 5.22 (d, *J* = 3.1 Hz, 1H), 4.54–4.43 (m, 1H), 4.41 (d, *J* = 7.9 Hz, 1H), 4.25 (dd, *J* = 11.8, 6.2 Hz, 1H), 3.22 (t, *J* = 9.1 Hz, 1H), 3.16 (t, *J* = 4.3 Hz, 1H), 3.06–2.85 (m, 2H), 2.42–2.17 (m, 2H), 2.04 (dd, *J* = 15.3, 11.5 Hz, 1H) ppm.

^13^C NMR (101 MHz, DMSO-*d*_6_) δ 206.1, 166.2, 155.0, 147.5, 145.9, 138.9, 126.3, 126.0, 119.6, 118.2, 115.5, 108.8, 102.3, 76.6, 74.1, 73.6, 70.3, 67.4, 63.7, 32.8, 28.2 ppm.

### 4.4. NMR Conditions

NMR spectra were recorded in DMSO-*d*_6_ at room temperature on a Varian Mercury 400 spectrometer (Varian Inc., Palo Alto, CA, USA) equipped with ATB PFG probe head using standard 5 mm Wilmad^®^ NMR tubes (Merck, Budapest, Hungary). The pulse programs were taken from the vendors software library (VnmrJ 3.1). As chemical shift reference, the residual solvent signals were used (DMSO-*d*_6_ at 2.500 ppm in ^1^H and 39.520 in ^13^C).

### 4.5. UHPLC-DAD-HR-MS/MS Analyses

To analyze the quantitative composition of *J. nigra* samples, we used a Dionex Ultimate 3000 UHPLC system (3000RS diode array detector, TCC-3000RS column thermostat, HPG-3400RS pump, SRD-3400 solvent rack degasser, WPS-3000TRS autosampler) (Thermo Fischer Scientific, Waltham, MA, USA), hyphenated with an Orbitrap^®^ Q Exactive Focus Mass Spectrometer equipped with an electrospray ionization source (Thermo Fischer Scientific). For the chromatographic separation of the constituents, a Kinetex C18 (75 × 3 mm i.d., 2.6 μm; Phenomenex) column was used as stationary phase (maintained at 25 °C). Mobile phase: 0.1% formic acid in water (eluent A) and a mixture of 0.1% formic acid in water and acetonitrile (20:80, *v*/*v*) (eluent B). Gradient elution was as follows: 5–100% B (0.0–12.0 min), 100% B (12.0–13.5 min), 100–5% B (13.5–14.0 min), 5% B (14.0–15.5 min), flow rate: 0.3 mL/min. The ESI source was operated in negative ionization mode and operation parameters were optimized automatically using the built-in software (Thermo Scientific Xcalibur 4.1). The working parameters were as follows: spray voltage 2500 V; capillary temperature 320 °C; sheath gas (N_2_), 47.5 °C; auxillary gas (N_2_) 11.25 arbitrary units, spare gas (N_2_) 2.25 arbitrary units. The resolution of the full scan was 70,000, the scanning range was between *m*/*z* 100–1000 units. The most intense ions detected in full scan spectrum were selected for data-dependent MS/MS scan at a resolving power of 35,000, in the range of *m*/*z* 100–1000. Parent ions were fragmented with normalized collision energy of 10%, 30%, and 45%.

### 4.6. Quantitative UHPLC-DAD Conditions

Quantities of the isolated compounds in the PAMPA experiments (**14**, **58**, **74**, **86**, **92**, **106**, **141**, and **143**) were determined by UHPLC-DAD. The *Juglans* extracts were analyzed by an ACQUITY UPLC H-Class PLUS System equipped with a quaternary solvent delivery pump (QSM), an auto-sampler manager (FTN), a column compartment (CM), and a photodiode array (PDA) detector (Waters Corporation). An Acquity BEH C18 column (100 × 2.1 mm i.d., 1.7 µm; Waters Corporation) maintained at 30 °C was used as stationary phase. Eluent A was 0.3% acetic acid in water and eluent B was acetonitrile, the following gradient elution was applied (flow rate: 0.3 mL/min): 5.0–100.0% B (0.0–11.0 min), 100.0–5.0% B (11.0–11.5 min), 5% B (11.5–15.0 min). The injection volume was 5 µL and chromatograms were recorded at 200–400 nm. The chromatograms acquired at the UV absorption maxima of each compound were used for data evaluation.

### 4.7. Validation of the Quantitative Method

#### 4.7.1. Preparation of Standard Solutions, Linearity, and Selectivity

Quantitation was performed by the external standard method. Stock solutions containing 10 mM of the isolated compounds (**14**, **58**, **143**, **74**, **86**, **106**, **141**, **92**) in HPLC-grade methanol were prepared. For the preparation of the calibration curve, stock solutions were diluted with 50% methanol of HPLC-grade and 50% distilled water, to yield solutions with concentrations of 0.78125, 1.5625, 3.125, 6.25, 12.5, 25, 50, and 100 μM. Each standard solution was prepared in triplicate and injected once. Standard solutions were stored at 4 °C before injection. Linearity curves were constructed by plotting peak areas against corresponding concentrations. Slope, intercept, and correlation coefficient were determined by least squares polynomial regression analysis. Limits of detection (LOD) and quantitation (LOQ) were determined at signal-to-noise (S/N) ratios 3 and 10, respectively. The selectivity of the method was evaluated by analyzing blank samples (HPLC-grade methanol). The linearity regression equations, correlation coefficients (r^2^), linearity ranges, and LOD and LOQ values of the method are shown in Supplementary Table S1.

#### 4.7.2. Precision, Accuracy, and Repeatability

Quality control samples were prepared at 6.25, 25, and 100 μM nominal concentrations. All samples were prepared in triplicate and injected once on the same day (intra-day precision and accuracy), or on three consecutive days (inter-day precision and accuracy). Precision as relative standard deviation (RSD%) and accuracy as mean percent recovery (%) were calculated using Equations (1) and (2):

(1) $$Precision\;(RSD\%)=\left(\frac{Standard\;Deviation}{Mean\;Concentration}\right)\times 100$$

(2) $$Accuracy\;(\%)=\left(\frac{{Concentration}_{experimental}}{{Concentration}_{nominal}}\right)\times 100$$

Retention time repeatability was assessed by injecting the standard solutions in six successive parallels. Intra-day and inter-day precision and accuracy data evaluated at low, mid, and high concentration ranges are shown in Supplementary Table S2.

### 4.8. Evaluation of the In Vitro Activity of the Isolated Compounds

#### Cell Culturing and Evaluation of In Vitro Cytostasis on Carcinoma Cell Lines

The cytostatic effect of the compounds was studied on tumor cell cultures in vitro. MDA-MB-231 human breast adenocarcinoma [95] cells were cultured in DMEM medium supplemented with 10% FBS, 2 mM L-glutamine, penicillin–streptomycin antibiotics mixture (50 IU/mL, and 50 μg/mL, respectively), 1 mM sodium pyruvate, and 1% non-essential amino acid mixture. A2058 human melanoma cells [96], and HT-29 colorectal carcinoma cells [97] were cultured in RPMI medium supplemented with 10% FBS, 2 mM L-glutamine, and penicillin–streptomycin antibiotics mixture (50 IU/mL and 50 μg/mL, respectively). Cell lines were generous gifts from Dr. József Tóvári (Department of Experimental Pharmacology, National Institute of Oncology, Budapest, Hungary).

For the selectivity study, a non-human primate cell culture was used. Vero E6 cells were established from kidney tissue sampled from an African green monkey (*C. sabaeus*) They originated from a primary culture initiated in March 1962 by Yoshihiro Yasumura at Chiba University in Japan [98]. Vero E6 cells were obtained from the European Collection of Authenticated Cell Cultures (ECACC 85020206) and maintained in DMEM high-glucose (4.5 g/L) medium (Lonza, Basel, Switzerland) containing 10% FBS (Gibco, Thermo Fisher Scientific) and supplemented with 2 mM of L-glutamine (Lonza), 1 mM sodium pyruvate (Merck), and CellCultureGuard (PanReacApplichem, ITW Reagents, Darmstadt, Germany) at 37 °C in a humidified atmosphere of 5% CO_2_.

All cell cultures were maintained at 37 °C in a humidified atmosphere with 5% CO_2_. The cells were grown to confluence and then divided into 96-well tissue culture plates with the initial cell number of 5.0 × 10^3^ cells/well. After 24 h of incubation at 37 °C, the cells were treated with the compounds in 200 μL final volume containing 1.0 *v*/*v* % DMSO at 0.16–100 μM (daunomycin: 0.016–10 μM, tamoxifen: 0.16–100 μM) concentration overnight. Control cells were treated with serum-free medium or DMSO (c = 1.0 *v*/*v* %) at the same conditions. After this incubation period, cells were washed twice with serum-free medium, and following that, they were cultured for another 72 h in a 10% serum-containing medium at 37 °C. After that, cell viability was determined using the Alamar Blue assay. Alamar Blue is a non-toxic, resazurin-based dye that living cells reduce to a fluorescent molecule, resorufin [99]. Resazurin sodium salt (Merck, Darmstadt, Germany) was dissolved in PBS at c = 0.15 mg/mL, pH 7.4). A measure of 22 uL of the dye was added to each well and incubated at 37 °C for 3 h until the pink color of the reduced dye appeared. Fluorescence intensity in each well was measured using a Synergy H4 multimode microplate reader (BioTek, Winooski, VT); at λ_ex_ = 530/30 and λ_em_ = 610/10 nm. The cytostatic effect (%) was calculated with a high degree of precision using the following Equation (3):

(3) $$cytostatic\;effect\;(\%)=\left[1-\left(\frac{{Fluorescence\;intensity}_{treated}}{{Fluorescence\;intensity}_{control}}\right)\right]\times 100$$

The cytostasis values were expressed in the percentage of untreated control. The 50 percent inhibitory concentration (IC_50_) was determined with a rigorous approach by fitting a sigmoid curve on the data points using Microcal™ Origin2021 software and then calculating X values at Y = 50. These values were expressed in micromolar units, providing a precise measure of the compound’s effectiveness.

### 4.9. Parallel Artificial Membrane Permeability Assay (PAMPA)

A parallel artificial membrane permeability assay (PAMPA) was used to determine the effective permeability (*P_e_*) for the isolated compounds **14**, **58**, **74**, **86**, **92**, **106**, **141**, and **143**. Stock solutions (10 mM in methanol) were diluted with the defined buffer (pH 7.4 for the PAMPA-BBB and pH 6.8 for the PAMPA-GI assays) to obtain the donor solutions (composition: 594.0 μL buffer + 6.0 μL stock solution). The buffers were prepared as follows: pH = 6.8: 20.2 g Na_2_HPO_4_ ∙ 7H_2_O and 3.4 g NaH_2_PO_4_ ∙ H_2_O dissolved in distilled water to achieve the final volume of 1000.0 mL, pH adjustment with 0.5 M NaOH or 0.5 M HCl; pH = 7.4: one PBS tablet (Phosphate Buffered Saline, pH 7.4; Sigma Aldrich) dissolved in 200.0 mL distilled water. Donor solutions were filtered through Phenex-RC 15 mm, 0.2 μm syringe filters (Gen-Lab Ltd., Budapest, Hungary).

For the PAMPA-BBB test, 5 μL of porcine polar brain lipid extract (PBLE) solution (16.0 mg PBLE + 8.0 mg cholesterol dissolved in 600.0 μL *n*-dodecane) was applied for each well of the 96-well polycarbonate-based filter donor plates (top plate) (Multiscreen™-IP, MAIPN4510, pore size 0.45 μm; Merck). For the PAMPA-GI assay, the wells of the top plate were coated with 5 μL of the mixture of 16.0 mg phosphatidylcholine + 8.0 mg cholesterol dissolved in 600.0 μL *n*-dodecane. A measure of 150.0 μL of aliquots of the filtrated donor solutions were placed on the membrane. The 96-well PTFE acceptor plates (bottom plates) (Multiscreen Acceptor Plate, MSSACCEPTOR; Merck), were filled with 300.0 μL buffer solution (0.01 M PBS buffer, pH 7.4). The donor plate was placed upon the acceptor plate, and both plates were incubated together at 37 °C for 4 h in a Heidolph Titramax 1000 Vibrating platform shaker (Heidolph, Schwabach, Germany).

After incubation, sandwich plates were separated and the concentrations of each compound in the starting donor solution and in the acceptor and donor wells were determined in triplicate by UHPLC-DAD method described above. UV spectra and chromatograms were recorded at 200–400 nm and the chromatograms acquired at the UV absorption maxima of each compound were used for data evaluation. The effective permeability and the membrane retention in the PAMPA-BBB and the PAMPA GI experiments were calculated by Equations (4)–(7), respectively [100]:

(4) $$P_{e}=\frac{-2.303}{A(t-\tau_{SS})}\cdot \left(\frac{V_{A}\cdot V_{D}}{V_{A}+V_{D}}\right)\cdot lg\left[1-\left(\frac{V_{A}+V_{D}}{\left(1-MR\right)\cdot V_{D}}\right)\times \left(\frac{C_{A}\left(t\right)}{C_{D}\left(0\right)}\right)\right]$$

(5) $$P_{e}=\frac{-2.303}{A(t-\tau_{SS})}\cdot \left(\frac{1}{1+r_{a}}\right)\cdot lg\left[-r_{a}+\left(\frac{1+r_{a}}{1-MR}\right)\times \left(\frac{C_{D}\left(t\right)}{C_{D}\left(0\right)}\right)\right]$$

where *P_e_* is the effective permeability coefficient (cm/s), *A* is the filter area (0.24 cm^2^), *V_D_* and *V_A_* are the volumes in the donor (0.15 cm^3^) and acceptor phases (0.30 cm^3^), *t* is the incubation time (s), *τ*_*SS*_ is the time (s) to reach steady-state (240 s), *C_D_*(*t*) is the concentration (mol/cm^3^) of the compound in the donor phase at time *t*, *C_D_*(0) is the concentration (mol/cm^3^) of the compound in the donor phase at time 0, and MR is the estimated membrane retention factor (the estimated mole fraction of solute lost to the membrane), *r_a_* is the sink asymmetry ratio (gradient-pH-induced), defined as:

(6) $$r_{a}=\frac{V_{D}}{V_{A}}\times \frac{P_{e}^{\left(A\to D\right)}}{P_{e}^{\left(D\to A\right)}}$$

(7) $$MR=1-\frac{C_{D}\left(t\right)}{C_{D}\left(0\right)}-\frac{V_{A}}{V_{D}}\frac{C_{A}\left(t\right)}{C_{D}\left(0\right)}$$

All experiments were performed in three triplicates on three consecutive days (n = 9), caffeine standard was used as positive, while rutin as negative control. *C*log*P* values were calculated by ChemAxon Marvin 22.3.

## Figures and Tables

**Table 1:** UHPLC-MS/MS data and tentative characterization of constituents from *Juglans nigra* leaf, bark, and pericarp extracts.

No.	Tentative Characterization	t_R_ (min)	[M−H]^−^ (*m*/*z*)	Fragment Ions (*m*/*z*)	Presence of Compounds ^a^									References
					JnLC	JnLE	JnLM	JnBC	JnBE	JnBM	JnPC	JnPE	JnPM	
**1**	caffeoyl-*O*-hexose	1.05	341	387 [M+HCOOH−H]^−^, 377 [M+Cl]^−^, 179, 135, 119	+	+	+	+	+	+	+	+	+	[38]
**2**	malic acid	1.10	133	125, 115, 105, 99, 89, 75, 73	+		+			+	+	+	+	[39,40]
**3**	monogalloyl-hexose	1.11	331	271, 211, 169, 125					+	+				[41]
**4**	monogalloyl-hexose	1.21	331	271, 241, 211, 169, 125, 113, 107						+		+	+	[41]
**5**	monogalloyl-hexose	1.33	331	271, 241, 211, 169, 125, 107			+		+				+	[41]
**6**	mono-HHDP-hexose	1.41	481	301, 275, 257, 247, 229, 203				+	+	+		+	+	[42]
**7**	monogalloyl-hexose	1.60	331	271, 211, 169			+		+	+		+	+	[41]
**8**	mono-HHDP-hexose	1.71	481	301, 275, 257, 249, 229, 203				+	+	+		+	+	[42]
**9**	dihydroxy-naphthoquinone	1.73	189	379 [2M−H]^−^, 173, 117			+					+	+	[43]
**10**	monogalloyl-hexose	1.85	331	663 [2M−H]^−^, 271, 241, 211, 169, 151, 139, 125, 123			+		+	+		+	+	[41]
**11**	monogalloyl-hexose	2.01	331	271, 211, 169, 125									+	[41]
**12**	monogalloyl-hexose	2.19	331	271, 211, 169, 125			+		+	+		+	+	[41]
**13**	monogalloyl-dihexose	2.49	493	331, 271									+	[44]
**14**	gallic acid ^b^	2.50	169	339 [2M−H]^−^, 125, 113			+		+	+		+	+	[38]
**15**	monogalloyl-hexose	2.64	331	271, 211, 169, 125			+		+	+		+	+	[41]
**16**	monogalloyl-dihexose ^c^	2.74	493	465, 331, 271, 211, 169						+			+	[44]
**17**	digalloyl-hexose	2.81	483	465, 331, 313, 271, 211, 169			+		+	+		+	+	[42]
**18**	linear diarylheptanoid hexoside ^c^	2.86	493	331, 313, 283			+			+			+	[16,38]
**19**	monogalloyl-dihexose	2.93	493	313, 301, 271, 169, 125			+			+			+	[44]
**20**	dihydroxybenzoyl-*O*-hexoside	3.28	315	153, 152, 108			+		+	+		+	+	[38,41]
**21**	A-type procyanidin dimer ^c^	3.30	575	401, 309, 287, 243, 169, 135, 125, 107			+			+			+	[45]
**22**	bis-HHDP-hexose	3.31	783	301, 275					+	+		+	+	[46,47]
**23**	methylgalloyl-*O*-hexose ^c^	3.37	345	331, 271, 211, 183	+	+	+	+	+	+			+	[38,42]
**24**	digalloyl-hexose	3.39	483	465, 331, 313, 271, 211, 169			+		+	+		+	+	[42]
**25**	galloyl-HHDP-hexose	3.53	633	483, 301, 275, 169					+	+			+	[46,48]
**26**	bis-HHDP-hexose	3.61	783	633, 481, 391, 305, 301, 291, 281, 275, 273 257, 229, 201, 193, 175, 173, 169, 161, 125								+	+	[46,47]
**27**	unknown	3.66	337	299, 179, 174, 147, 133					+	+			+	
**28**	bis-HHDP-hexose	3.68	783	633, 481, 391, 305, 301, 291, 281, 275, 273 257, 229, 201, 193, 175, 173, 169, 161, 125					+	+				[46,47]
**29**	digalloyl-hexose	3.77	483	465, 331, 313, 271, 211, 169			+		+	+		+	+	[42]
**30**	3-*O*-caffeoylquinic acid	3.81	353	707 [2M−H]^−^, 191, 179, 135	+	+	+							[49,50]
**31**	galloyl-HHDP-hexose	3.84	633	481, 463, 301, 275, 273, 169, 125					+	+		+	+	[46,48]
**32**	galloyl-HHDP-DHHDP-hexose ^c^	3.90	951	783, 633, 483, 475, 391								+		[48]
**33**	hydroxy-dimethoxybenzoyl-*O*-hexose ^c^	3.92	359	719 [2M−H]^−^, 197, 182			+	+	+	+		+	+	[38,51]
**34**	monogalloyl-pentose	4.01	301	415 [M+TFA−H]^−^, 275, 271, 241, 211, 169, 139, 125			+			+		+	+	[38]
**35**	monogalloyl-hexose	4.02	331	663 [2M−H]^−^, 241, 169			+	+	+					[41]
**36**	galloyl-HHDP-DHHDP-hexose	4.03	951	783, 633, 483, 475, 391								+	+	[48]
**37**	hydroxy-dimethoxybenzoyl-*O*-hexoside ^c^	4.10	359	719 [2M−H]^−^, 197, 182						+		+	+	[38,51]
**38**	bis-HHDP-hexose	4.13	783	481, 391, 301, 275, 273, 257, 249, 169, 125					+	+		+	+	[46,47]
**39**	galloyl-HHDP-DHHDP-hexose	4.21	951	783, 673, 483, 301, 275, 239, 169								+	+	[48]
**40**	trigalloyl-hexose	4.28	635	465, 313, 271, 169, 125									+	[41,48]
**41**	3-*O*-coumaroylquinic acid	4.29	337	675 [2M−H]^−^, 191. 163, 119			+							[49,50]
**42**	galloyl-HHDP-DHHDP-hexose	4.29	951	783, 673, 483, 301, 275, 239, 169					+					[48]
**43**	digalloyl-HHDP-hexose	4.34	785	633, 453, 301, 275, 249								+	+	[47,52]
**44**	galloyl-HHDP-hexose	4.38	633	463, 301, 275, 249, 169, 151					+	+		+	+	[46,48]
**45**	hydroxy-dimethoxybenzoyl-*O*-hexoside ^c^	4.39	359	719 [2M−H]^−^, 197, 182	+	+	+		+	+				[38,51]
**46**	gallotannin	4.42	829	414 [M−2H]^2−^, 673, 483, 423, 363, 301, 275, 217, 210, 183			+		+	+		+	+	[41,42]
**47**	5-*O*-caffeoylquinic acid	4.44	353	375 [M+Na−2H]^−^,, 191, 179, 173, 135	+		+							[49,50]
**48**	trigalloyl-hexose	4.48	635	465, 313, 271, 169, 125			+							[41,48]
**49**	digalloyl-HHDP-hexose	4.56	785	635, 467, 465, 301, 275, 249, 183, 169			+							[47,52]
**50**	trihydroxy-tetralone-*O*-hexoside isomer ^c^	4.57	355	295, 235, 193, 175, 174, 165, 160, 147, 145, 131							+	+	+	[53,54]
**51**	gallotannin	4.60	451	565 [M+TFA−H]^−^, 313, 193, 169, 125				+	+	+				[41,42]
**52**	trihydroxy-tetralone-*O*-hexoside isomer ^c^	4.62	355	401 [M+HCOOH−H]^−^, 193, 175, 131, 113			+					+	+	[53,54]
**53**	methylgallic acid	4.63	183	169, 168, 139, 137, 127, 123	+	+	+	+	+	+			+	[38,51]
**54**	trigalloyl-hexose	4.63	635	483, 331, 271, 169								+	+	[41,48]
**55**	digalloyl-HHDP-hexose	4.68	785	635, 467, 465, 301, 275, 249			+							[47,52]
**56**	galloyl-HHDP-hexose	4.72	633	463, 301, 275, 169	+									[46,48]
**57**	trigalloyl-hexose	4.72	635	465, 313, 271, 169, 125					+	+				[41,48]
**58**	ethylgallic acid ^b^	4.73	197	169, 125					+	+		+	+	[38,51]
**59**	gallotannin ^c^	4.75	925	835, 785, 635, 509, 505, 489, 477, 467, 457, 301, 275, 179, 169, 151, 125			+		+	+				[41,42]
**60**	digalloyl-HHDP-hexose	4.78	785	635, 467, 465, 301, 275, 249			+		+	+		+	+	[47,52]
**61**	trigalloyl-hexose	4.83	635	483, 465, 331, 313, 301, 271, 211, 169, 125			+				+	+	+	[41,48]
**62**	trigalloyl-hexose	4.89	635	483, 465, 331, 313, 301, 271, 211, 169, 125					+	+		+	+	[41,48]
**63**	linear diarylheptanoid pentoside ^c^	4.94	463	313, 207, 175, 149							+	+	+	[16,38]
**64**	hydroxy-naphthyl-*O*-hexoside ^c^	4.97	321	367 [M+HCOOH−H]^−^, 213, 201, 158					+	+		+	+	[55]
**65**	monogalloyl-dihexose	4.98	493	391, 313, 301, 271, 211		+	+	+	+	+	+	+	+	[44]
**66**	trigalloyl-hexose	5.03	635	483, 465, 313, 301, 275, 271, 211, 169			+	+	+	+	+	+	+	[41,48]
**67**	gallocatechin-*O*-gallate/epigallocatechin-*O*-gallate ^c^	5.08	457	339, 331, 305, 169, 125	+		+		+	+	+	+	+	[56]
**68**	digalloyl-HHDP-hexose	5.05	785	831 [M+HCOOH−H]^−^, 635, 301, 275, 169			+		+	+		+	+	[47,52]
**69**	galloyl-methylgallic acid isomer	5.08	335	183, 168									+	[42]
**70**	digalloylshikimic acid	5.09	477	313, 169, 125		+	+							[42]
**71**	gallotannin ^c^	5.14	925	835, 785, 635, 509, 489, 467, 457, 301, 275, 179, 169, 151, 125			+		+	+				[41,42]
**72**	ellagitannin (castalagin) derivative ^c^	5.15	965	933, 445, 301								+	+	[38,46]
**73**	galloyl-bis-HHDP-hexose ^c^	5.18	935	785, 663, 551, 467, 451, 301, 275							+	+	+	[48]
**74**	quercetin-3-*O*-xyloside (reynoutrin) ^b^	5.21	433	301, 300		+	+	+	+	+	+	+	+	[38]
**75**	hydrojuglone-*O*-hexoside	5.26	337	675 [2M−H]^−^, 451 [M+TFA−H]^−^, 175, 174, 173, 145, 131		+	+	+	+	+	+	+	+	[31,53,57]
**76**	galloyl-bis-HHDP-hexose ^c^	5.28	935	785, 633, 467, 433, 301, 275, 203, 175, 169					+	+	+			[48]
**77**	myricetin-3-*O*-hexoside	5.31	479	317, 316		+	+	+	+	+			+	[39]
**78**	tetragalloyl-hexose ^c^	5.32	787	635, 465, 313, 301, 271, 169			+		+	+			+	[48]
**79**	galloyl-bis-HHDP-hexose ^c^	5.41	935	636 [M−2H]^2−^, 897, 787, 643, 467, 463, 393					+	+	+	+	+	[48]
**80**	quercetin-3-*O*-hexosyl-deoxyhexoside	5.45	609	301, 300, 271, 255	+		+		+	+	+	+	+	[38]
**81**	trigalloyl-HHDP-hexose ^c^	5.46	937	787, 491, 468, 393, 301, 275, 169			+				+		+	[48,58]
**82**	galloyl-bis-HHDP-hexose ^c^	5.47	935	785, 655, 633, 493, 467, 391, 301, 275, 169					+					[48]
**83**	trihydroxy-tetralone	5.51	193	307 [M+TFA−H]^−^, 175, 149, 113			+	+	+	+	+	+	+	[53]
**84**	galloyl-bis-HHDP-hexose ^c^	5.54	935	785, 655, 633, 491, 301, 275, 169					+	+	+			[48]
**85**	tetragalloyl-hexose ^c^	5.63	787	635, 617, 483, 465, 331, 313, 301, 275, 169			+		+	+		+	+	[48]
**86**	myricetin-3-*O*-rhamnoside (myricitrin) ^b^	5.64	463	927 [2M−H]^−^, 317, 316, 179, 151		+	+	+	+	+	+	+	+	[38]
**87**	trihydroxy-dimethoxyflavone ^c^	5.69	329	314, 299, 284, 195, 165, 149							+		+	[59]
**88**	ellagic acid hexoside ^c^	5.69	463	301		+	+	+	+	+	+	+	+	[38,51]
**89**	tetragalloyl-hexose ^c^	5.69	787	635, 617, 465, 313, 301, 169, 125			+		+	+				[48]
**90**	quercetin-3-*O*-hexoside	5.73	463	927 [2M−H]^−^, 301, 300, 271, 255	+	+	+	+	+	+	+	+	+	[39]
**91**	trigalloyl-HHDP-hexose ^c^	5.75	937	787, 491, 468, 393, 301, 275, 169			+	+		+		+	+	[48,58]
**92**	1,2,3,4-tetrahydro-7,8-dihydroxy-4-oxonaphthalen-1-yl-6-*O*-galloyl-glucoside ^b,c^	5.77	507	621 [M+TFA−H]^−^, 331, 271, 211, 169, 125		+	+		+	+	+	+	+	[53]
**93**	catechin-gallate/epicatechin-gallate ^c^	5.82	441	289, 195, 169, 150, 125289, 245, 229, 169, 125					+	+	+	+	+	[60]
**94**	pentagalloyl-hexose	5.82	939	787, 469, 335, 183			+			+		+		[41,48]
**95**	galloylquinic acid derivative	5.83	573	525, 482, 391, 377, 343, 329, 195, 181, 165							+	+	+	[49]
**96**	galloyl-HHDP-DHHDP-hexose ^c^	5.85	951	933, 507, 469, 271, 211, 331			+							[48]
**97**	trigalloyl-HHDP-hexose ^c^	5.87	937	787, 657, 301, 275, 169					+	+	+			[48,58]
**98**	pentagalloyl-hexose	5.88	939	787, 469, 335, 316, 213, 183, 167			+				+			[41,48]
**99**	tetramethoxyflavone derivative ^c^	5.92	567	341, 326, 311, 179, 119						+		+	+	[59]
**100**	dihydroxy-methoxyflavanone-*O*-hexosyl-deoxyhexoside ^c^	5.94	593	285				+		+				[61]
**101**	tetragalloyl-hexose ^c^	5.94	787	635, 617, 465, 447, 301, 211, 169, 125					+	+				[48]
**102**	pentagalloyl-hexose	5.99	939	787, 769, 635, 617, 469, 301, 275, 169, 125			+		+	+	+		+	[41,48]
**103**	tetrahydroxy-methoxyflavone-*O*-hexuronoside ^c^	6.00	491	315, 300				+	+	+	+	+	+	[62]
**104**	unknown	6.07	361	343, 179, 165, 145				+	+	+				
**105**	pentagalloyl-hexose	6.10	939	769, 617, 169, 469, 440, 425, 416, 388, 376, 297, 295, 236, 227, 214, 205, 194, 113			+	+	+	+	+			[41,48]
**106**	quercetin-3-*O*-rhamnoside (quercitrin) ^b^	6.12	447	895 [2M−H]^−^, 561 [M+TFA−H]^−^, 493 [M+HCOOH−H]^−^, 301, 300, 271, 255, 179	+	+	+	+	+	+	+	+	+	[38]
**107**	galloyl-methylgallic acid isomer	6.14	335	449 [M+TFA−H]^−^, 183			+		+	+			+	[42]
**108**	unknown	6.15	391	373, 193, 183, 179, 175				+	+	+	+			
**109**	trihydroxy-methoxychalcone-*O*-hexoside	6.24	447	285, 165, 119				+	+	+				[31]
**110**	caffeoyl–feruloyltartaric acid	6.25	487	325, 324		+	+		+	+		+	+	[63]
**111**	galloyl-methylgallic acid isomer	6.25	335	671 [2M−H]^−^, 183						+				[42]
**112**	tetramethoxyflavone-*O*-deoxyhexoside ^c^	6.30	487	341, 326, 311, 271								+	+	[59,64]
**113**	pentagalloyl-hexose	6.37	939	787, 735, 683, 635, 487, 301, 169			+		+	+				[41,48]
**114**	hexahydroxy-methoxyflavone ^c^	6.39	347	461 [M+TFA−H]^−^, 249, 227, 187, 243, 229, 215, 201, 173, 145, 113							+	+		[59]
**115**	rosmarinic acid	6.44	359	719 [2M−H]^−^, 197, 179, 161, 135	+									[65]
**116**	myricetin-3-*O*-galloyl-deoxyhexoside isomer ^c^	6.45	615	463, 317, 179, 169, 151, 137, 125		+	+	+	+	+	+	+	+	[66,67]
**117**	myricetin-3-*O*-galloyl-deoxyhexoside isomer ^c^	6.54	615	317, 179, 169, 151		+	+	+	+	+	+	+	+	[66,67]
**118**	unknown	6.55	673	511, 347, 329, 317, 316, 169								+	+	
**119**	linear diarylheptanoid pentoside ^c^	6.55	463	331, 313, 161			+		+	+		+	+	[16,38]
**120**	kaempferol-3-*O*-deoxyhexoside ^c^	6.56	431	863 [2M−H]^−^, 545 [M+TFA−H]^−^, 285, 284, 255, 227, 161	+	+	+	+	+	+	+	+	+	[38]
**121**	tetrahydroxy-methoxyflavanone-*O*-hexoside ^c^	6.69	479	317, 165, 151				+	+	+				[68]
**122**	pentagalloyl-hexose	6.70	939	787, 635, 617, 331, 301, 169, 125				+						[41,48]
**123**	kaempferol-3-*O*-galloyl-hexoside ^c^	6.74	599	437, 285					+	+				[67,69]
**124**	digalloylshikimic acid	6.79	477	313, 183, 169, 125		+	+							[42]
**125**	gallotannin ^c^	6.83	673	657, 631, 630, 493, 478, 301, 275, 169, 125								+	+	
**126**	quercetin-3-*O*-galloyl-deoxyhexoside isomer ^c^	6.91	599	301, 179, 169, 151		+	+	+	+	+	+	+	+	[67,69]
**127**	gallotannin ^c^	7.03	517	631 [M+TFA−H]^−^, 539[M+Na−2H]^−^, 469, 301, 175				+	+	+				
**128**	quercetin-3-*O*-galloyl-deoxyhexoside isomer ^c^	7.02	599	301, 179, 169, 151		+	+	+	+	+		+	+	[67,69]
**129**	hydrojuglone	7.10	175	113				+	+	+				[57]
**130**	dihydroxy-methoxyflavanone-*O*-hexoside	7.31	447	561 [M+TFA−H]^−^, 285, 165, 119				+	+	+				[31,70]
**131**	trihydroxy-methoxyflavanone-*O*-hexoside ^c^	7.38	463	577 [M+TFA−H]^−^, 509 [M+HCOOH−H]^−^, 301, 165, 135				+	+	+				
**132**	caffeoylquinic acid shikimate isomer ^c^	7.39	509	615 [M+TFA−H]^−^, 353, 347, 346, 329, 317, 161				+	+		+	+	+	[71]
**133**	caffeoylquinic acid shikimate isomer ^c^	7.59	509	615 [M+TFA−H]^−^, 353, 347, 346, 329, 317, 173, 161		+			+		+	+	+	[71]
**134**	diarylheptanoid aglycone ^c^	7.72	343	179, 167, 165, 164, 135, 121, 119				+	+	+	+	+	+	
**135**	oxo-dihydroxy-octadecenoic acid ^c^	7.82	327	373 [M+HCOOH−H]^−^, 311, 229, 221, 211, 193, 189, 183, 171, 167	+	+	+	+	+	+	+	+	+	[72]
**136**	caffeoylquinic acid shikimate isomer ^c^	7.90	509	353, 347, 329, 317, 173, 171, 161				+	+	+	+	+	+	[71]
**137**	caffeoylquinic acid shikimate isomer ^c^	8.00	509	353, 347, 329, 317, 173, 171, 161				+	+	+	+	+	+	[71]
**138**	diarylheptanoid aglycone ^c^	8.16	329	193, 171, 139, 135, 121, 119		+	+	+	+	+	+			
**139**	trihydroxy-octadecenoic acid ^c^	8.25	329	473, 357, 329, 313, 281, 229, 211, 171, 139	+	+	+	+	+	+	+	+	+	[72]
**140**	juglanin G ^c^	8.73	341	297, 269, 267, 237, 217, 183, 182									+	
**141**	juglone ^b^	8.76	173	145, 154, 128, 117, 111							+	+		[31,38,54,55]
**142**	trihydroxy-octadecanoic acid ^c^	9.05	329	314, 267, 249, 207, 193, 165, 135, 119	+	+	+	+	+	+	+	+	+	[71]
**143**	ellagic acid ^b^	9.06	301	165, 153	+	+	+	+	+	+	+	+	+	[38]
**144**	unknown	9.08	619	473, 301, 165, 135						+				
**145**	juglanin B ^c^	9.31	327	313, 312, 295, 294, 272, 254, 253, 249, 241, 239, 225, 221, 211, 207, 201, 195, 189, 183	+	+	+	+	+	+	+	+	+	[43,55]
**146**	epicatechin/catechin derivative ^c^	9.48	345	363 [M+Na−2H]^−^, 319, 317, 301, 289, 245, 189, 175, 161						+	+		+	
**147**	unknown	9.77	327	221, 206, 153, 135, 121				+		+				
**148**	unknown	9.79	347	329, 327, 305, 303, 223, 221				+	+			+		
**149**	unknown	10.01	285	165, 155, 119, 113			+	+	+	+				
**150**	trihydroxy-binaphthalene-tetrone ^c^	10.04	361	343, 333, 317, 316, 289, 273, 261, 249, 233			+				+	+		[55]
**151**	diarylheptanoid aglycone ^c^	10.07	293	236, 221, 220, 205, 177, 164, 155, 148, 113				+	+	+	+	+	+	
**152**	unknown	10.33	325	311, 310, 253, 249, 213, 183, 167, 155, 113	+	+	+	+	+	+				
**153**	unknown	10.39	293	265, 255, 249, 209, 207, 205, 189, 167, 155, 147, 119							+	+	+	
**154**	secoisolariciresol ^c^	10.48	361	343, 333, 317, 316, 289, 273, 261, 249, 233							+	+	+	[53]
**155**	unknown	10.97	293	249, 193							+	+	+	
**155**	epicatechin/catechin derivative ^c^	11.61	345	367 [M+Na−2H]^−^, 317, 301, 289, 273, 261, 249, 245, 197, 155, 141							+	+		
**156**	bisjuglone isomer ^c^	11.62	345	367, 317, 301, 289, 273, 261, 249, 245, 197, 155							+	+		[55]
**157**	unknown	11.64	293	285, 265, 167, 155, 113	+	+	+	+	+	+				
**158**	unknown	11.85	293	275, 171, 155, 121			+	+	+	+				
**159**	bisjuglone isomer ^c^	12.02	345	317, 301, 289, 273, 261, 249, 245, 155							+	+		[55]
**160**	unknown	12.65	295	277, 265, 249, 171, 155, 113	+	+	+	+	+	+	+			
**161**	trisjuglone ^c^	13.46	515	537 [M+Na−2H]^−^, 487, 471, 459, 443, 415, 401, 387, 379, 249, 155							+	+		[55]

^a^ Abbreviations: JnLC: *J. nigra* leaf chloroform extract; JnLE: *J. nigra* leaf ethyl acetate extract; JnLM: *J. nigra* leaf methanol extract; JnBC: *J. nigra* bark chloroform extract; JnBE: *J. nigra* bark ethyl acetate extract; JnBM: *J. nigra* bark methanol extract; JnPC: *J. nigra* pericarp chloroform extract; JnPE: *J. nigra* pericarp ethyl acetate extract; JnPM: *J. nigra* pericarp methanol extract; +: present in the extract; HHDP: hexahydroxydiphenoyl; DHHDP: dehydrohexahydroxydiphenoyl; TFA: trifluoroacetic acid; HCOOH: formic acid; ^b^ Compared to a reference substance; ^c^ Reported for the first time in *J. nigra.*

**Table 2:** HR-MS data of the isolated constituents of *J. nigra* pericarp.

Compound	[M−H]^−^ (*m*/*z*) Experimental	[M−H]^−^ (*m*/*z*) Calculated	Error (ppm)	Molecular Formula	Fragment ions (*m*/*z*)
**gallic acid (14)**	169.01321	169.01315	0.060	C_7_H_6_O_5_	125.02304 (C_6_H_5_O_3_)
**ethyl gallate (58)**	197.04501	197.04445	0.560	C_9_H_10_O_5_	169.0129 (C_7_H_5_O_5_)
**myricetin-3-*O*-rhamnoside (** **86)**	463.08853	463.087102	1.428	C_21_H_20_O_12_	317.02808 (C_15_H_9_O_8_), 316.02249 (C_15_H_8_O_8_), 271.02472 (C_14_H_7_O_6_), 178.99770 (C_8_H_3_O_5_)
**1,2,3,4-tetrahydro-7,8-dihydroxy-4-oxonaphthalen-1-yl-6-*O***- **galloyl-glucoside (92)**	507.11368	507.113317	0.363	C_23_H_24_O_13_	331.0676(C_13_H_15_O_10_), 271.0464 (C_11_H_11_O_8_), 211.02445 (C_9_H_7_O_6_), 169.0134 (C_7_H_5_O_5_), 125.0232 (C_6_H_5_O_3_)
**quercetin-3-*O*-rhamnoside (** **106)**	447.09314	447.092188	0.952	C_21_H_20_O_11_	301.03458 (C_15_H_9_O_7_), 300.02753 (C_15_H_8_O_7_), 271.02484 (C_14_H_7_O_6_), 255.02982 (C_14_H_7_O_5_)
**ellagic acid (143)**	300.99902	300.997894	1.126	C_14_H_6_O_8_	-
**juglone (141)**	173.02351	173.023321	0.189	C_10_H_6_O_3_	154.97263 (C_10_H_3_O_2_), 145.02817 (C_9_H_5_O_2_), 126.88013 (C_9_H_3_O), 116.92687 (C_8_H_5_O)
**quercetin-3-*O*-xyloside (** **74)**	433.04187	433.07653	−4.668	C_20_H_18_O_11_	300.9992 (C_15_H_9_O_7_)

**Table 3:** The in vitro cytostatic activity of the compounds on different cell cultures.

Compound	IC_50_ (µM)			
	**Cell Culture**			
	**MDA-MB 231**	**A2058**	**HT-29**	**VERO E6** **Non Tumorous**
**quercetin-3-*O*-rhamnoside (** **106)**	>100	>100	>100	15.9 ± 0.7
**ellagic acid (143)**	>100	>100	>100 (33.0% inhibition at 100 µM)	>100
**myricetin-3-*O*-rhamnoside (** **86)**	>100	>100	>100	3.7 ± 0.7
**juglone (141)**	9.9 ± 0.7	13.5 ± 0.7	0.53 ± 0.1	3.7 ± 0.7
**1,2,3,4-tetrahydro-7,8-dihydroxy-4-oxonaphthalen-1-yl-6-*O***- **galloyl-glucoside (92)**	>100	>100	>100	61.1 ± 5.3
**gallic acid (14)**	49.8 ± 3.5	57.2 ± 4.7	71.2 ± 7.9	49.9 ± 2.8
**ethyl gallate (58)**	>100	102.5 ± 0.7 (56.4% inhibition at 100 µM)	>100	82.1 ± 5.7
**quercetin-3-*O*-xyloside (** **74)**	>100	>100	>100	>100
***Daunomycin***	*0.7* ± 0.1	*0.9* ± 0.1	*0.2* ± 0.05	*1.1* ± 0.05
***Tamoxifen***	*3.4* ± 0.7	*1.0* ± 0.2	*n.d.*	*3.5* ± 0.6

**Table 4:** Results of the PAMPA experiments expressed as log*P_e_* values (*n* = 9).

Compound	log*P_e_* PAMPA-BBB (*n* = 9)	log*P_e_* PAMPA-GI (*n* = 9)
**gallic acid (14)**	n.d.	n.d.
**ethyl gallate (58)**	−5.77 ± 0.31	−5.43 ± 0.26
**myricetin-3-*O*-rhamnoside** **(86)**	n.d.	n.d.
**1,2,3,4-tetrahydro-7,8-dihydroxy-4-oxonaphthalen-1-yl-6-*O***- **galloyl-glucoside (92)**	n.d.	n.d.
**quercetin-3-*O*-rhamnoside** **(106)**	n.d.	n.d.
**ellagic acid (143)**	−6.65 ± 0.50	−6.13 ± 0.61
**juglone (141)**	−4.11 ± 0.19	−4.41 ± 0.10
**quercetin-3-*O*-xyloside** **(74)**	n.d.	n.d.

Abbreviations: n.d.: not detected in the acceptor phase; PAMPA-GI: parallel artificial membrane permeability assay for the gastrointestinal tract; PAMPA-BBB: parallel artificial membrane permeability assay for the blood–brain barrier.

## Data Availability

Data is contained within the article and Supplementary Materials.

## Supplementary Materials

The following supporting information can be downloaded at: https://www.mdpi.com/article/10.3390/ijms25136930/s1.
