# Supplementary material for: Comprehensive Characterization of Phytochemical Composition, Membrane Permeability, and Antiproliferative Activity of Juglans nigra Polyphenols
Source: Int J Mol Sci. 2024 Jun 25;25(13):6930. doi: 10.3390/ijms25136930 (PMC11241769; doi:10.3390/ijms25136930)
Supplement: Supplementary file 1 [file ijms-25-06930-s001.zip › ijms-3062602-supplementary.pdf]

Supplementary Material

Comprehensive Characterization of Phytochemical Composition, Membrane Permeability, and Antiproliferative Activity of *Juglans nigra* Polyphenols

Rita Osztie [1], Tamás Czeglédi [1], Sarah Ross [2], Bence Stipsicz [3,4], Eszter Kalydi [5], Szabolcs Béni [6], Imre Boldizsár [1,7], Eszter Riethmüller [1], Szilvia Bősze [4,8] and Ágnes Alberti [1,*]

[1] Department of Pharmacognosy, Semmelweis University, Üllői út 26, 1085 Budapest, Hungary;
osztie.rita@stud.semmelweis.hu (R.O.); czegledi.tamas@semmelweis.hu (T.C.); boldizsar.imre@semmelweis.hu (I.B.); riethmuller.eszter@semmelweis.hu (E.R.)

[2] Department Pharmaceutical Biology, Institute for Drug Discovery, University of Leipzig,
Eilenburger Str. 14, 04317 Leipzig, Germany; sarah.ross@studserv.uni-leipzig.de

[3] Institute of Biology, Doctoral School of Biology, ELTE Eötvös Loránd University, Pázmány Péter sétány 1/C,
1117 Budapest, Hungary; stipsicz@student.elte.hu

[4] HUN-REN-ELTE Research Group of Peptide Chemistry, Hungarian Research Network, ELTE Eötvös Loránd University, Pázmány Péter sétány 1/A, 1117 Budapest, Hungary; szilvia.bosze@ttk.elte.hu

[5] Institute of Organic Chemistry, Semmelweis University, Hőgyes Endre u. 7., 1092 Budapest, Hungary;
kalydi.eszter@semmelweis.hu

[6] Institute of Chemistry, ELTE Eötvös Loránd University, Pázmány Péter sétány 1/A, 1117 Budapest, Hungary; szabolcs.beni@ttk.elte.hu

[7] Department of Plant Anatomy, Institute of Biology, ELTE Eötvös Loránd University, Pázmány Péter sétány 1/C, 1117 Budapest, Hungary

[8] Department of Genetics, Cell- and Immunobiology, Semmelweis University, Nagyvárad tér 4.,
1089 Budapest, Hungary

[*] Correspondence: alberti.agnes@semmelweis.hu (Á.A.)

Table of contents

**No.**	**Content**
**Figure S1.**	UHPLC-DAD chromatograms of *J. nigra* pericarp extracts (max plot); chloroform extract (green); ethyl acetate extract (red); methanol extract (black). Compound numbers refer to Table 1.
**Figure S2.**	UHPLC-DAD chromatograms of *J. nigra* bark extracts (max plot); chloroform extract (green); ethyl acetate extract (red); methanol extract (black). Compound numbers refer to Table 1.
**Figure S3.**	UHPLC-DAD chromatograms of *J. nigra* leaf extracts (max plot); chloroform extract (green); ethyl acetate extract (red); methanol extract (black). Compound numbers refer to Table 1.
**Figure S4.**	Atom numbering of compound **92** for the chemical shift assignment. The annotation is arbitrary and not equivalent to that used in compound nomenclature.
**Figure S5.**	^{1}H NMR spectrum of compound **92** (400 MHz, 298 K, DMSO-d_6)
**Figure S6.**	^{13}C NMR spectrum of compound **92** (100 MHz, 298 K, DMSO-d_6)
**Table S1.**	Method validation: regression, LOQ and LOD of the quantitative UHPLC-DAD method
**Table S2.**	Method validation: Precision and accuracy of the quantitative UHPLC-DAD method

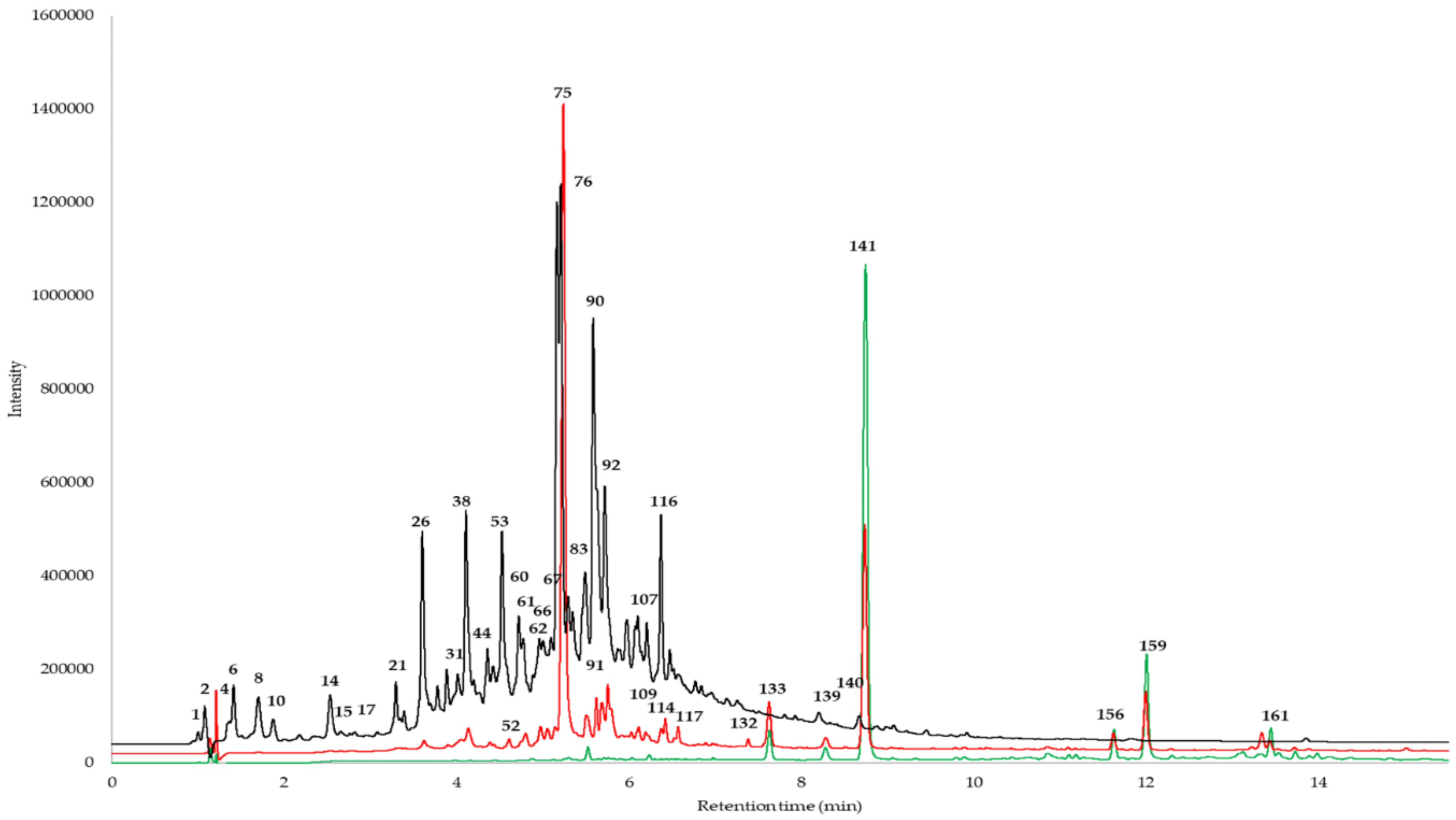

Figure S1. UHPLC-DAD chromatograms of *J. nigra* pericarp extracts (max plot); chloroform extract (green); ethyl acetate extract (red); methanol extract (black). Compound numbers refer to Table 1.

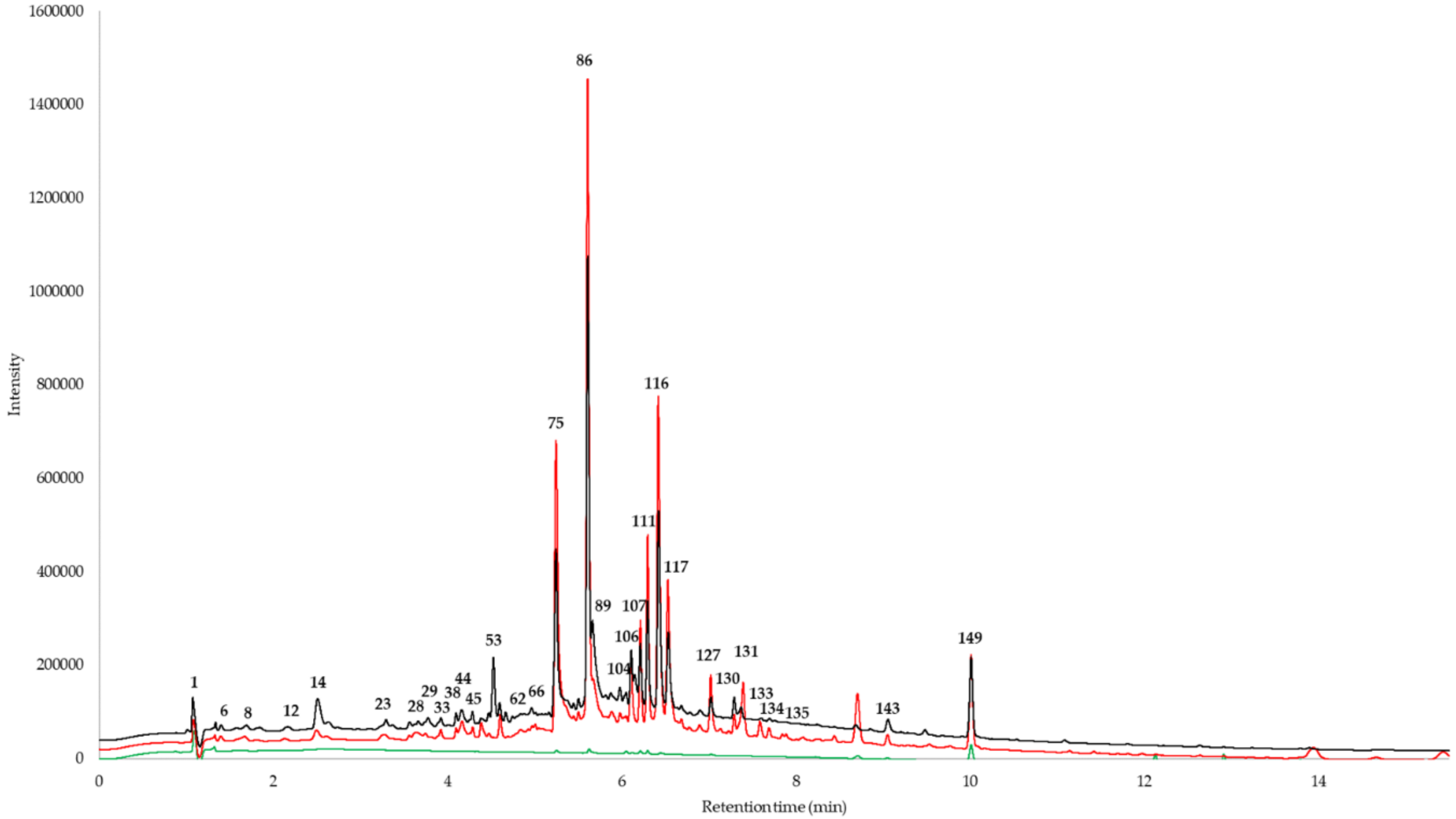

Figure S2. UHPLC-DAD chromatograms of *J. nigra* bark extracts (max plot); chloroform extract (green); ethyl acetate extract (red); methanol extract (black). Compound numbers refer to Table 1.

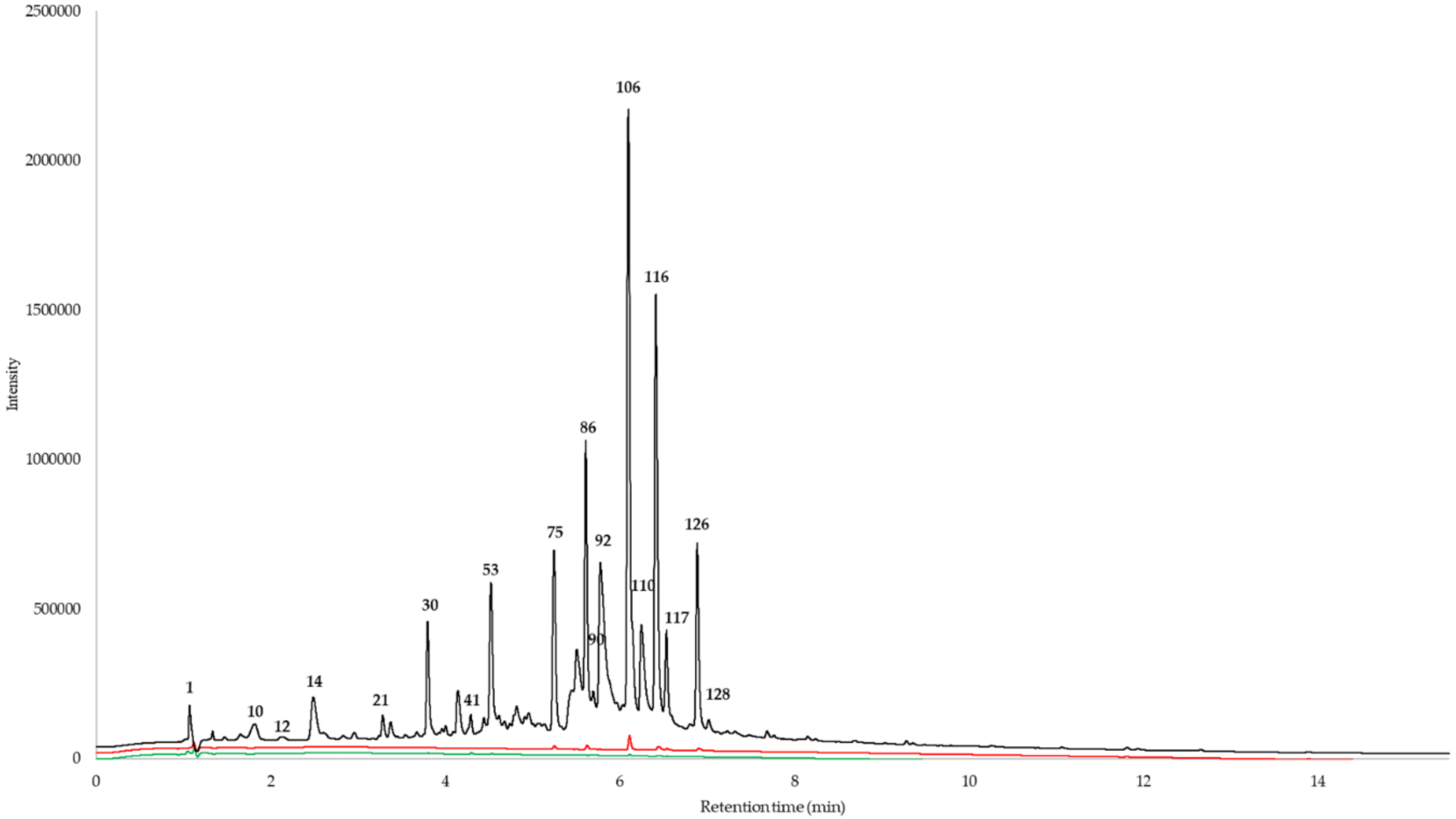

Figure S3. UHPLC-DAD chromatograms of *J. nigra* leaf extracts (max plot); chloroform extract (green); ethyl acetate extract (red); methanol extract (black). Compound numbers refer to Table 1.

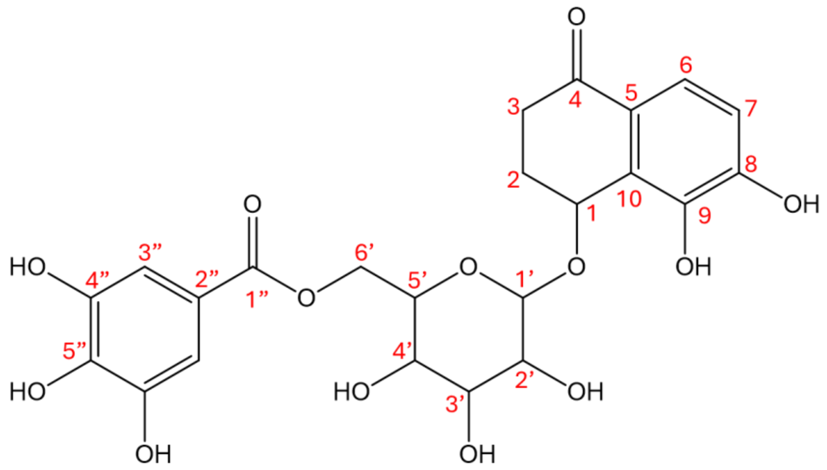

^{1}H NMR (400 MHz, DMSO-d_6) δ 7.15 (d, *J* = 9.0 Hz, 1H, H-6), 6.98 (s, 2H, H-3''), 6.81 (d, *J* = 9.0 Hz, 1H, H-7), 5.22 (d, *J* = 3.1 Hz, 1H, H-1), 4.54 – 4.43 (m, 1H, H-6'a), 4.41 (d, *J* = 7.9 Hz, 1H, H-1'), 4.25 (dd, *J* = 11.8, 6.2 Hz, 1H, H-6'b), 3.50-3.42 (m, overlapped with water, 1H, H-5'), 3.22 (t, *J* = 9.1 Hz, 1H, H-3'), 3.16 (t, J = 4.3 Hz, 1H, H-4'), 3.06 – 2.85 (m, 2H, H-3a, H-2'), 2.42 – 2.17 (m, 2H, H-2a, H-3b), 2.04 (dd, *J* = 15.3, 11.5 Hz, 1H, H-2b) ppm.

^{13}C NMR (101 MHz, DMSO-d_6) δ 206.1 (C-4), 166.2 (C-1''), 155.0 (C-8), 147.5 (C-9), 145.9 (C-4''), 138.9 (C-5''), 126.3 (C-6), 126.0 (C-10), 119.6 (C-2''), 118.2 (C-7), 115.5 (C-5), 108.8 (C-3''), 102.3 (C-1'), 76.6 (C-4), 74.1 (C-5), 73.6 (C-2), 70.3 (C-3), 67.4 (C-1), 63.7 (C-6'), 32.8 (C-3), 28.2 (C-2) ppm.

Figure S4. Atom numbering of compound **92** for the chemical shift assignment. The annotation is arbitrary and not equivalent to that used in compound nomenclature.

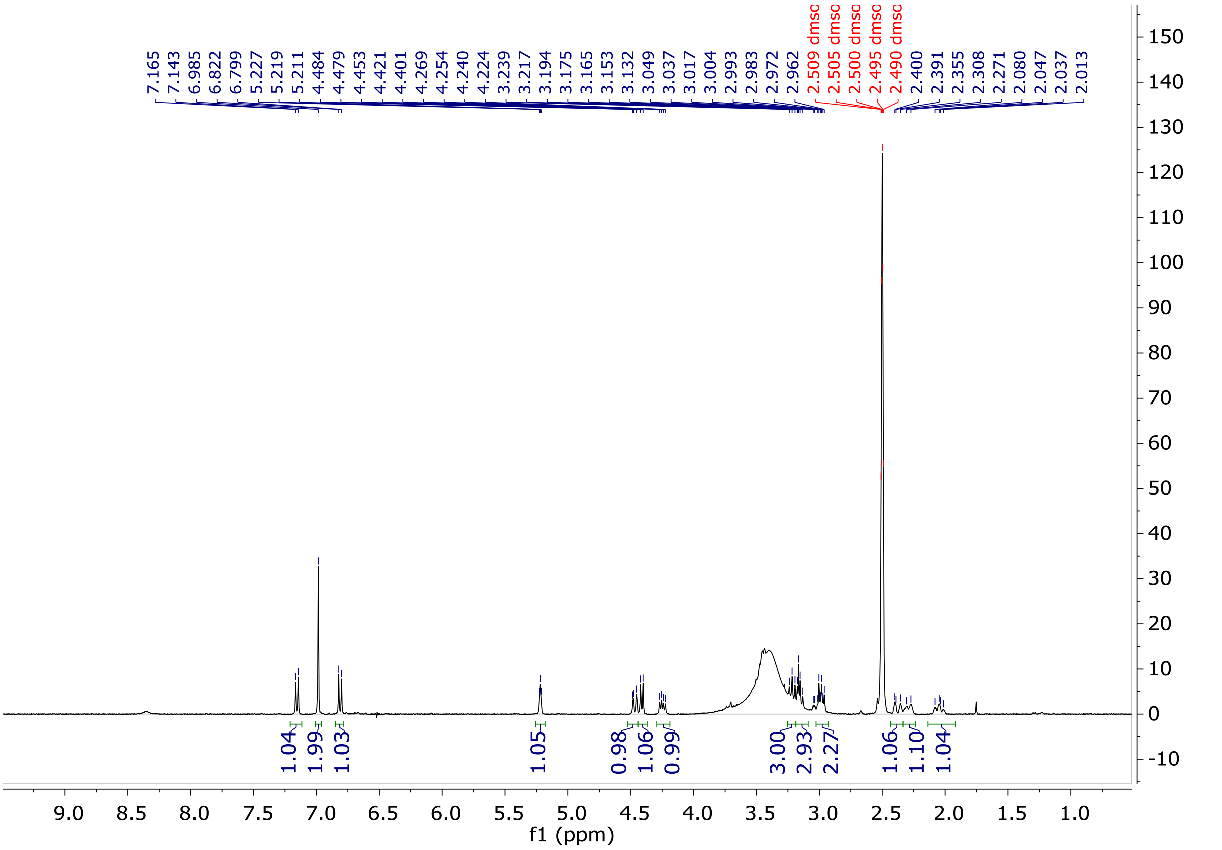

Figure S5. ^{1}H NMR spectrum of compound **92** (400 MHz, 298 K, DMSO-*d*6)

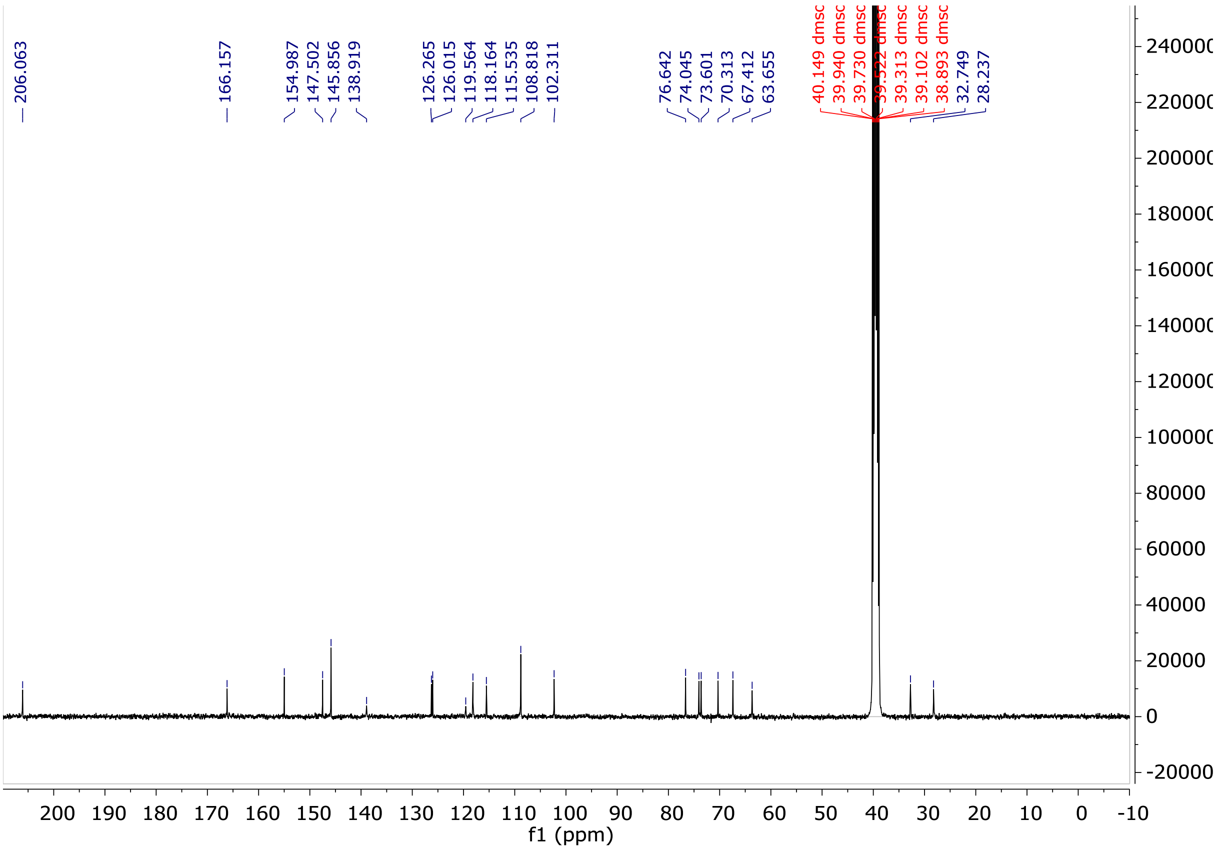

Figure S6. ^{13}C NMR spectrum of compound **92** (100 MHz, 298 K, DMSO-*d*6)

Table S1. Method validation: regression, LOQ and LOD of the quantitative method

Compound	Regression equation	r^2	Regression range (µg/mL) [1]	LOD µg/mL)	LOQ (µg/mL)
**14**	y = 421.88x - 35.681	1.0000	0.13281-17	0.082	0.273
**58**	y = 78.609x	0.9991	0.07735-19.8	0.047	0.158
**141**	y = 43.20x + 9,975	0.9984	0.3625-46.4	0.211	0.704
**92**	y = 37.176x	0.9993	0.3969-50.8	0.323	1.076
**106**	y = 528.87x	0.9986	0.35-44.8	0.467	1.559
**143**	y = 1429.20x – 102.17	0.9998	0.11797-30.2	0.0076	0.025
**141**	y = 428.80x + 36.413	0.9997	0.1359-17.4	0.3165	1.055
**74**	y = 64.769x	0.9990	0.339-43.4	0.2177	0.726

[1] Corresponding to 1.5625 – 100 µM

Table S2. Method validation: Precision and accuracy of the quantitative method

Nominal conc. (μM)	Precision (RSD%)		Accuracy (%)	
	Intra-day	Inter-day	Intra-day	Inter-day
**14**				
6.25	1.29	9.76	99.65	77.26
25	1.27	9.85	100.93	86.19
100	0.85	3.97	100.01	94.27
**58**				
6.25	1.01	6.12	81.55	82.92
25	1.28	2.52	92.99	90.74
100	1.72	5.39	101.63	98.22
**86**				
6.25	3.49	7.66	95.27	88.62
25	1.65	9.70	96.52	117.32
100	0.55	4.23	105.88	100.95
**92**				
6.25	3.21	5.40	91.45	95.88
25	0.92	4.60	91.71	95.70
100	0.25	0.74	99.68	100.35
**106**				
6.25	0.27	6.39	103.82	110.24
25	0.14	3.89	105.35	109.22
100	0.10	2.66	106.58	109.24
**143**				
6.25	0.96	9.71	96.46	90.41
25	1.12	9.27	99.11	94.49
100	0.15	9.96	99.62	94.85
**141**				
6.25	1.09	4.96	99.77	97.28
25	4.25	9.50	97.78	105.27
100	8.75	9.06	100.53	102.01
**74**				
6.25	2.03	7.00	82.49	73.30
25	0.56	0.50	89.74	91.10
100	0.20	7.03	99.43	90.10
